# Genome-wide analysis of major intrinsic proteins in the tree plant *Populus trichocarpa*: Characterization of XIP subfamily of aquaporins from evolutionary perspective

**DOI:** 10.1186/1471-2229-9-134

**Published:** 2009-11-20

**Authors:** Anjali Bansal Gupta, Ramasubbu Sankararamakrishnan

**Affiliations:** 1 Department of Biological Sciences and Bioengineering, Indian Institute of Technology Kanpur, Kanpur 208016, India

## Abstract

**Background:**

Members of major intrinsic proteins (MIPs) include water-conducting aquaporins and glycerol-transporting aquaglyceroporins. MIPs play important role in plant-water relations. The model plants *Arabidopsis thaliana*, rice and maize contain more than 30 MIPs and based on phylogenetic analysis they can be divided into at least four subfamilies. *Populus trichocarpa *is a model tree species and provides an opportunity to investigate several tree-specific traits. In this study, we have investigated *Populus *MIPs (PtMIPs) and compared them with their counterparts in *Arabidopsis*, rice and maize.

**Results:**

Fifty five full-length MIPs have been identified in *Populus *genome. Phylogenetic analysis reveals that *Populus *has a fifth uncharacterized subfamily (XIPs). Three-dimensional models of all 55 PtMIPs were constructed using homology modeling technique. Aromatic/arginine (ar/R) selectivity filters, characteristics of loops responsible for solute selectivity (loop C) and gating (loop D) and group conservation of small and weakly polar interfacial residues have been analyzed. Majority of the non-XIP PtMIPs are similar to those in *Arabidopsis*, rice and maize. Additional XIPs were identified from database search and 35 XIP sequences from dicots, fungi, moss and protozoa were analyzed. Ar/R selectivity filters of dicots XIPs are more hydrophobic compared to fungi and moss XIPs and hence they are likely to transport hydrophobic solutes. Loop C is longer in one of the subgroups of dicot XIPs and most probably has a significant role in solute selectivity. Loop D in dicot XIPs has higher number of basic residues. Intron loss is observed on two occasions: once between two subfamilies of eudicots and monocot and in the second instance, when dicot and moss XIPs diverged from fungi. Expression analysis of *Populus *MIPs indicates that *Populus *XIPs don't show any tissue-specific transcript abundance.

**Conclusion:**

Due to whole genome duplication, *Populus *has the largest number of MIPs identified in any single species. Non-XIP MIPs are similar in all four plant species considered in this study. Small and weakly polar residues at the helix-helix interface are group conserved presumably to maintain the hourglass fold of MIP channels. Substitutions in ar/R selectivity filter, insertion/deletion in loop C, increasing basic nature of loop D and loss of introns are some of the events occurred during the evolution of dicot XIPs.

## Background

Water transport in different parts of a plant is significantly contributed by the integral membrane channel protein, aquaporin, which is a member of the Major Intrinsic Protein (MIP) superfamily [1]. In addition to their role in plant soil-water relations [2,3], members of this family are also implicated in plant reproduction [4,5], cell elongation [6], plant cell osmoregulation [7] and seed germination [8]. Aquaporins also influence leaf physiology and leaf movements [9,10], drought resistance [11], salt tolerance [12,13] and fruit ripening [14] in plants. MIP family consists of both aquaporins [15] and aquaglyceroporins [16,17]. A large number of MIP genes have been identified in plants and they seem to be diverse. *Arabidopsis *[18], maize [19] and rice [20,21] each have more than 30 MIP genes. Phylogenetic analysis reveals that the MIP genes can be largely divided into at least four different subfamilies and they have been classified as plasma membrane intrinsic proteins (PIPs), tonoplast intrinsic proteins (TIPs), nodulin-26 intrinsic proteins (NIPs) and small basic intrinsic proteins (SIPs) [18,19,21,22]. Three additional subfamilies have been recently reported. In the nonvascular moss *Physcomitrella patens *which is a primitive land plant, a novel plant MIP (GIP) homologous to bacterial glycerol channels found in gram-positive bacteria has been identified [23]. Two other subfamilies found recently in the same species are hybrid intrinsic proteins (HIPs) and unrecognized X intrinsic proteins (XIPs) [24]. Substrate specificity, expression and localization of many members of PIPs, TIPs and NIPs have been investigated. Plant MIPs localize in plasma membranes (PIPs and some NIPs) [25-27], tonoplast (TIPs) [28], endoplasmic reticulum (SIPs) [29] and other subcellular compartments [30]. In addition to water and glycerol [31-33], PIPs, TIPs and NIPs facilitate the transport of other unconventional neutral solutes and gases [34]. This includes urea [35-37], lactic acid [38] and metalloids like boron [27,39], silicon [26], arsenic and antimony [40,41]. Carbon dioxide [42], hydrogen peroxide [43] and NH3 [44,45] are among the other molecules that are transported by plant MIPs. The transport activity of these MIP genes is regulated by many factors including cotranslational and post-translational modifications [46-48], gating [49] or subcellular trafficking [50,51]. Members of XIPs and HIPs are the least characterized and they need further investigation regarding solute transport, expression and other properties.

Three-dimensional structures of proteins belonging to MIP family have been determined from several organisms [52-58] including a plant aquaporin SoPIP2;1 from spinach [49]. All MIP structures exhibit a conserved hourglass fold with α-helical bundle comprising six transmembrane (TM) helices (H1 to H6) and two half-helices. The half-helices forming the seventh TM helix are from loops B and E (LB and LE) that also possess the signature sequence Asn-Pro-Ala (NPA). These conserved motifs from the two half-helices meet approximately at the center of the membrane giving rise to one of the two pore constrictions. The second constriction, also known as aromatic/arginine (ar/R) selectivity filter, is formed by four residues towards the extracellular side approximately 8 Å from the NPA region. The four residues in this selectivity filter are contributed by transmembrane helices H2, H5 and the loop LE. Molecular mechanism of water and glycerol transport, exclusion of charged groups and specificity of solute transport have been investigated by computational [59-63] and experimental studies [64,65]. Recently, homology modeling was carried out on *Arabidopsis*, rice and maize MIPs [21,66] and the structures were classified based on the residues in the ar/R selectivity filter. The diversity of pore configurations indicated that the plant MIPs could transport much more diverse solutes than their counterparts in mammals.

The genome sequence of the model tree plant *Populus trichocarpa *(Black cottonwood) has been recently determined [67]. Phylogenetically, *Populus *is more closely related to *Arabidopsis *than the model cereal plant rice. *Populus *is a eudicot and both *Populus *and *Arabidopsis *are clustered in angiosperm Euroside I clade [68]. The availability of genomes of *Arabidopsis*, *Populus *and rice will facilitate the study of comparative biology of all the three species. As a second eudicot genome sequence with its modest genome size, *Populus trichocarpa *offers unique opportunity to study some aspects that cannot be studied in other model annual plants [68]. Examples include wood development, seasonality, flowering and natural variation [69]. Apart from its genomic sequence, other *Populus *genomic resources such as *Populus *EST sequences, full-length cDNA sequences and DNA microarrays also offer tools to study *Populus *biology [70-72]. *Populus *is also a good model system in which long distance transport of water and nutrients can be investigated. However, there are only few studies on poplar aquaporins and their role in long distance transport of water and other nutrients. Seven aquaporins have been investigated in mycorrhized poplar plants and it has been shown that there is a strong increase in the capacity of water transport in plasma membrane of root cells [73]. Analysis of EST sequences from the root of hybrid cottonwood described the expression levels of *Populus *PIP and TIP members during different stages of adventitious root development [74]. A recent study by Danielson and Johanson [24] identified a group of aquaporins from *Populus *belonging to the unrecognized XIP category. As in *Arabidopsis *and rice, the availability of *Populus *genome sequence gives an opportunity to identify and characterize the whole repertoire of MIPs in this species. In this paper, we have carried out genome-wide analysis of *Populus *MIPs from its genomic sequence and characterized them. We have identified 55 full-length MIP genes in *Populus *and this is the largest number of MIP genes identified in any single species to date. We have compared several features of *Populus *MIPs with their counterparts in *Arabidopsis*, rice and maize. The unique features identified in *Populus *MIPs are discussed in this paper.

## Results

### MIP genes in *Populus *genome

The whole genome shotgun (WGS) sequence of *Populus trichocarpa *[67] available at NCBI [75] was searched using TBLASTN [76] for genes coding for MIPs. The initial query sequence from rice OsPIP2;1 resulted in identification of 41 *Populus *MIPs (PtMIPs). Five other query sequences representing PIP, TIP, NIP, SIP and XIP family members from the initial search results yielded additional MIP proteins. A list of more than 50 full-length MIP proteins from *Populus *WGS contigs was obtained (Table 1) after discarding those sequences with missing transmembrane regions or interrupted by a stop codon in the middle of the sequence as predicted by the program GeneMark [77,78]. The *Populus *genome paper [67] has reported 67 genes belonging to major intrinsic protein family (Table S12 in the reference Tuskan et al. [67]), although the details are not mentioned. The Joint Genome Institute (JGI) has listed 63 aquaporin genes (KOG ID: 0223) belonging to *Populus trichocarpa*. We have carefully compared the MIP proteins from our TBLASTN search result with those 63 from JGI and found that there are 50 sequences common between both of them. We find that 9 of the 63 MIP proteins from JGI have to be discarded for various reasons (Additional file 1: Table S1). Four JGI sequences were not found in our search. One sequence from our search (NCBI accession no. AARH01008299) is not present in the JGI list. Thus, we have finally obtained 55 full-length MIP protein sequences from *Populus trichocarpa *which is the largest set of MIP sequences from any single species identified so far and they are listed in Table 1. The available data shows that forty four *Populus *MIP genes are nearly uniformly spread over 13 of the 19 haploid chromosomes. Nine out of 13 chromosomes have at least 3 MIPs each with the highest number of eight MIPs observed in chromosome IX (Table 1). The remaining genes are located on a scaffold not yet assigned to a chromosome.

**Table 1:** MIP genes in *Populus *genome identified from the NCBI whole genome shotgun contigs.

Name	NCBI WGS contig accession number	JGI accession number	EST accession	NPA motif (LB)^a^	NPA motif (LE)^a^	Chromosome location^e^
PtPIP1;1	AARH01004386	724520	DT472648	-	-	X

PtPIP1;2	AARH01003541	656216	BU868142	-	-	VIII

PtPIP1;3	AARH01001832	711735	DT474111	-	-	III

PtPIP1;4	AARH01003029	831918		-	-	VI

PtPIP1;5	AARH01006875	835561		-	-	XVI

PtPIP2;1	AARH01003794	821084	DT478685	-	-	IX

PtPIP2;2	AARH01002541	648808	DT473513	-	-	IV

PtPIP2;3	AARH01004412	567607	DT474367	-	-	X

PtPIP2;4	AARH01003539	563742	DT472264	-	-	VIII

PtPIP2;5	AARH01008299	826419	BU868919	-	-	*

PtPIP2;6^b^	AARH01008299	N/A	CV227359	-	-	*

PtPIP2;7	AARH01006751	735495	DT498815	-	-	XVI

PtPIP2;8	AARH01003912	821627		-	-	IX

PtPIP2;9^c^		836572		-	-	*

PtPIP2;10	AARH01008738	796664		-	-	*

PtTIP1;1	AARH01000429	549212		-	-	I

PtTIP1;2	AARH01003864	833283	DT499779	-	-	IX

PtTIP1;3	AARH01004405	822504	DT476627	-	-	X

PtTIP1;4	AARH01003540	656044	BU875073	-	-	VIII

PtTIP1;5	AARH01006799	667870	DT497619	-	-	XVI

PtTIP1;6	AARH01008323	589502	DT497300	-	-	*

PtTIP1;7	AARH01003929	558321		-	-	IX

PtTIP1;8	AARH01010693	828458		-	-	*

PtTIP2;1	AARH01000349	548890	DT496472	-	-	I

PtTIP2;2	AARH01001731	645978	DT488368	-	-	III

PtTIP2;3	AARH01001777	817166		-	-	III

PtTIP2;4	AARH01008349	676397		-	-	*

PtTIP3;1	AARH01010701	584517		-	-	*

PtTIP3;2	AARH01011328	811826		-	-	*

PtTIP4;1	AARH01003145	561759	CV233830	-	-	VI

PtTIP5;1^d^	AARH01001826	414059		-	-	III

PtTIP5;2^d^	AARH01008384	423803		-	-	*

PtNIP1;1^d^	AARH01002108	197507		-	-	IV

PtNIP1;2^d^	AARH01004854	235172		-	-	XI

PtNIP1;3	AARH01004327	566501		-	-	X

PtNIP1;4^c^		756079		-	-	II

PtNIP1;5^c^		754717		-	-	II

PtNIP2;1	AARH01007172	577637		-	-	XVII

PtNIP3;1	AARH01001861	757987		NPS	NPV	III

PtNIP3;2	AARH01000110	797136		NPS	NPV	I

PtNIP3;3	AARH01000968	708017	DT488082	-	-	I

PtNIP3;4	AARH01005007	823094		-	-	XI

PtNIP3;5	AARH01003691	803915		-	-	VIII

PtSIP1;1	AARH01005657	729942		NPT	-	XIII

PtSIP1;2	AARH01007966	665418	DT487219	NPT	-	XIX

PtSIP1;3	AARH01001280	755885		-	-	II

PtSIP1;4^c^		572968		-	-	XIX

PtSIP2;1	AARH01006621	734665	DN491635	NPL	-	XVI

PtSIP2;2	AARH01002935	652505		NPL	-	VI

PtXIP1;1	AARH01022440	829126		NPI	-	*

PtXIP1;2	AARH01003797	557139		NPI	-	IX

PtXIP1;3	AARH01002537	759781		NPI	-	IV

PtXIP1;4	AARH01003797	767334		NPL	-	IX

PtXIP1;5	AARH01003797	821124		-	-	IX

PtXIP2;1	AARH01003797	557138		SPV	-	IX

^a^Deviation of NPA signature motif in loops B and E is reported. '-' indicates that NPA motif is conserved.

^b^This gene is not listed in JGI. The coding regions in the NCBI WGS contig are 173376-173676, 173811-174106, 174224-174364, 174465-174584.

^c^Four PtMIP genes are not found in our TBLASTN search of NCBI database. Their JGI accession codes are given.

^d^The sequences for these genes predicted by GeneMark are longer than those found in JGI. GeneMark-predicted sequences have extensions in N- or C-terminus. In this study, we have used GeneMark predicted sequences due to their similarity with *Arabidopsis *sequences

^e^The symbol '*' indicates that these genes are located on a scaffold not yet assigned to a chromosome.

### Comparison of *Populus *MIPs with MIPs of *Arabidopsis*, rice and maize

**Figure 1:**
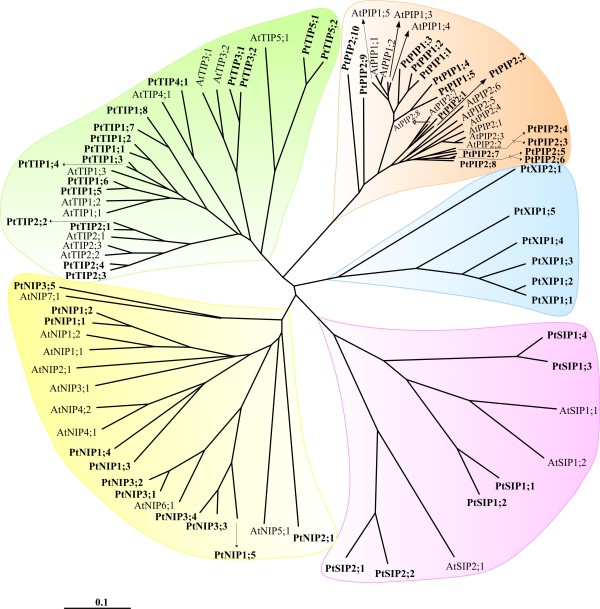
**Evolutionary relationship of *Populus *MIPs**. Phylogenetic analysis of all *Populus *MIPs is shown along with MIPs from *Arabidopsis*. Neighbor-Joining (NJ) method was used to create this unrooted tree. NJ method used the multiple sequence alignment generated by T-COFFEE to generate the tree. *Populus *MIP subfamilies PtPIPs, PtTIPs, PtNIPs and PtSIPs clustered with the corresponding *Arabidopsis *MIP subfamilies. XIPs observed only in *Populus *clearly form a separate group. Each MIP subfamily is shown with a specific background color to distinguish them from others. A similar result is obtained when the same analysis was carried out with rice and maize MIPs (Additional files 2 to 4).

PtMIPs were compared individually with MIPs from *Arabidopsis *(AtMIPs), rice (OsMIPs) and maize (ZmMIPs). Then all MIPs from the four plant species were compared together. Multiple sequence alignments of full length proteins using the program T-COFFEE [79] were generated on different sets of MIP sequences, namely (i) PtMIPs, (ii) PtMIPs and AtMIPs, (iii) PtMIPs and OsMIPs, (iv) PtMIPs and ZmMIPs and (v) PtMIPs, AtMIPs, OsMIPs and ZmMIPs. The trees created using these alignments by neighbor-joining (NJ) method shows that PtMIPs can be classified into five subfamilies. PIPs, TIPs, NIPs and SIPs from *Populus *clustered with the respective subfamilies from *Arabidopsis*, rice and maize (Figure 1, Additional files 2 to 4). The fifth subfamily belongs to the uncharacterized XIP family and is not observed in the other three plant species. Sequences belonging to neither HIP nor GIP family [23,24] are found in all the four plant species. When MIPs from all four plant species were considered together, the corresponding non-XIP subfamily members clustered together and XIPs observed only in *Populus *clustered separately (Additional file 4). The results of NJ method were found to be very similar to those by heuristic distance, parsimony and maximum likelihood methods with the clustering more or less maintained in all three methods (data not shown). Among the 55 PtMIPs, there are 15 PIPs, 17 TIPs, 11 NIPs, 6SIPs and 6XIPs. Both PIPs (15 PtPIPs vs 13 in other plants) and NIPs (11 PtNIPs vs. 9 to 13 in *Arabidopsis *and rice) are similar in number found in other plants. The expression of most of the PtPIP and PtTIP sequences are supported by the *Populus *EST sequences (Table 1). The increase in the number of PtMIPs can be attributed to the increase in the number of PtTIPs and PtSIPs and also the presence of a new XIP subfamily with 6 members. The other three plants have 10 to 11 TIPs and 2 to 3 SIPs. The additional 15 PtMIPs belonging to TIP, SIP and XIP subfamilies explain the largest number of MIPs observed in *Populus*.

Each subfamily was further subdivided into groups according to their clustering in the phylogenetic tree and their similarity with the known MIPs from other plants. As in other plants, *Populus *PIPs and TIPs have two (PtPIP1 and PtPIP2) and five (PtTIP1 to PtTIP5) subgroups respectively. However, maximum number of seven subgroups is observed for *Arabidopsis *NIPs while *Populus*, maize and rice NIPs have only three to four subgroups. Two PtNIP members (PtNIP3;1 and PtNIP3;2) have substitutions in both NPA motifs. Although, two subgroups are observed for PtSIP subfamily similar to other plants under study, the number of SIP proteins found in *Populus *is the maximum observed so far. The Ala residue in the first NPA motif in four out of 6 PtSIPs is substituted by Thr or Leu. The uncharacterized XIP family found only in *Populus *among the four species has two subgroups PtXIP1 and PtXIP2. While sequences from other subfamilies have been analyzed and studied experimentally, little is known about the XIP family members. We have identified additional members of XIP family and further sequence analysis and homology modeling helped us to characterize this subfamily further and the details are explained below.

### XIP subfamily members in other species

Danielson and Johanson [24] have reported 19 XIP members that included 5 *Populus *XIPs. Among the XIPs, 10 were from dicot plants other than *Populus*, three were from moss and one was from a protozoa. No XIP homolog was found in monocots. We examined all these sequences and found that the sequence from *Nicotiana benthamina *(GenBank ID: CK295158) lacks the first transmembrane segment. Similarly, one of the EST sequences for *Liriodendron tulipifera *(GenBank ID: DT60037) is lacking NCBI record. Hence, these two sequences were discarded for further analysis of XIP sequences. In addition to the 6 *Populus *XIPs identified in the present study (5 of them have been reported by Danielson and Johanson [24] also), we have considered the 12 additional XIP sequences from plants, moss and the protozoa reported earlier [24].

In order to identify additional XIP members, we used each of the six PtXIP sequence as a query and searched the plant EST databases using TBLASTN [80]. We have identified an additional 8 XIP sequences from dicot plants (Table 2). We also carried out TBLASTN searches on various completed and partial genome sequences of different organism groups available in NCBI. To our surprise, many hits were obtained from organisms that are classified as fungi with e-values ranging from 4.0E-17 to 1.0E-04. The program GeneMark [77,78] was used to identify the coding regions and we found 9 full-length (Table 3) and 5 partial fungi MIP sequences based on GeneMark predictions. Partial fungi sequences were not considered for further analysis (Additional file 1: Table S2). Thus our search of plant EST database and fungi genomic sequences yielded another additional 17 XIP sequences. Taken together, we have considered 6 *Populus *XIPs, 16 XIPs from other dicot plants, 9 fungi XIPs, 3 moss XIPs and 1 protozoan XIP (Total 35 XIPs) for further analysis.

**Table 2:** Additional dicot XIPs identified from TBLASTN search of plant EST databases.

XIP Name^a^	Species name	EST accession number	NPA motif (LB)^b^	NAP motif (LE)^b^
LsXIP1;1	*Lactuca sericola*	DW109344	-	-

CcXIP1;2	*Citrus climentina*	DY262747	NPL	-

CcXIP1;3	*Citrus climentina*	DY263320	NPL	-

CcXIP1;4	*Citrus climentina*	DY256802	NPL	-

CsXIP1;1	*Citrus sinensis*	CX049225	NPL	-

CsXIP1;2	*Citrus sinensis*	CX049224	NPL	-

RcXIP2;2	*Ricinus communis*	EG664940	SPT	-

RcXIP2;3	*Ricinus communis*	EG671494	SPA	-

^a^CcXIP1;1 and RcXIP2;1 are the XIPs from the respective species reported in Danielson and Johanson [24]. See also Figure 2.

^b^Deviation of NPA signature motif in loops B and E is reported. '-' indicates that NPA motif is conserved.

**Table 3:** XIPs identified from the genomic sequences of fungi

Name^a^	Species name	NCBI accession number *[Coding regions]*	NPA motif (LB)^b^	NPA motif (LE)^b^
F-PmXIP	*Penicillium marneffei*	ABAR01000036 *[47839-48091, 48166-48854]*	NPT	-

F-TsXIP	*Talaromyces stipitatus*	ABAS01000013 *[178746-178668, 178588-178022, 177946-177870, 177773-177696]*	NPT	-

F-TvXIP1	*Trichoderma virens*	ABDF01000006 *[317889-318119, 318215-318437, 318628-318715, 318809-319092, 319203-319360]*	NPM	NPS

F-TvXIP2	*Trichoderma virens*	ABDF01000215 *[85889-86154, 86313-86842, 86893-87062]*	-	-

F-FoXIP	*Fusarium oxysporum f. sp. Lycopersici*	AAXH01000716 *[22960-22681, 22584-21929]*	NPL	-

F-GmXIP	*Gibberella moniliformis*	AAIM02000133 *[47801-47522, 47424-46769]*	NPL	-

F-TaXIP	*Trichoderma atroviride*	ABDG01000060 *[27703-27452, 27334-27112, 26961-26874, 26815-26532, 26467-26289]*	NPT	-

F-TrXIP	*Trichoderma reesei*	AAIL01000183 *[17864-18130, 18289-18511, 18709-18796, 18890-19173, 19303-19460]*	NPM	NPS

F-AtXIP	*Aspergillus terreus*	AAJN01000055 *[25721-25970, 26017-26595, 26663-26751]*	SPT	-

^a^The names are prefixed with "F-" to distinguish fungi XIPs from other XIPs.

^b^Deviation of NPA signature motif in loops B and E is reported. '-' indicates that NPA motif is conserved.

**Figure 2:**
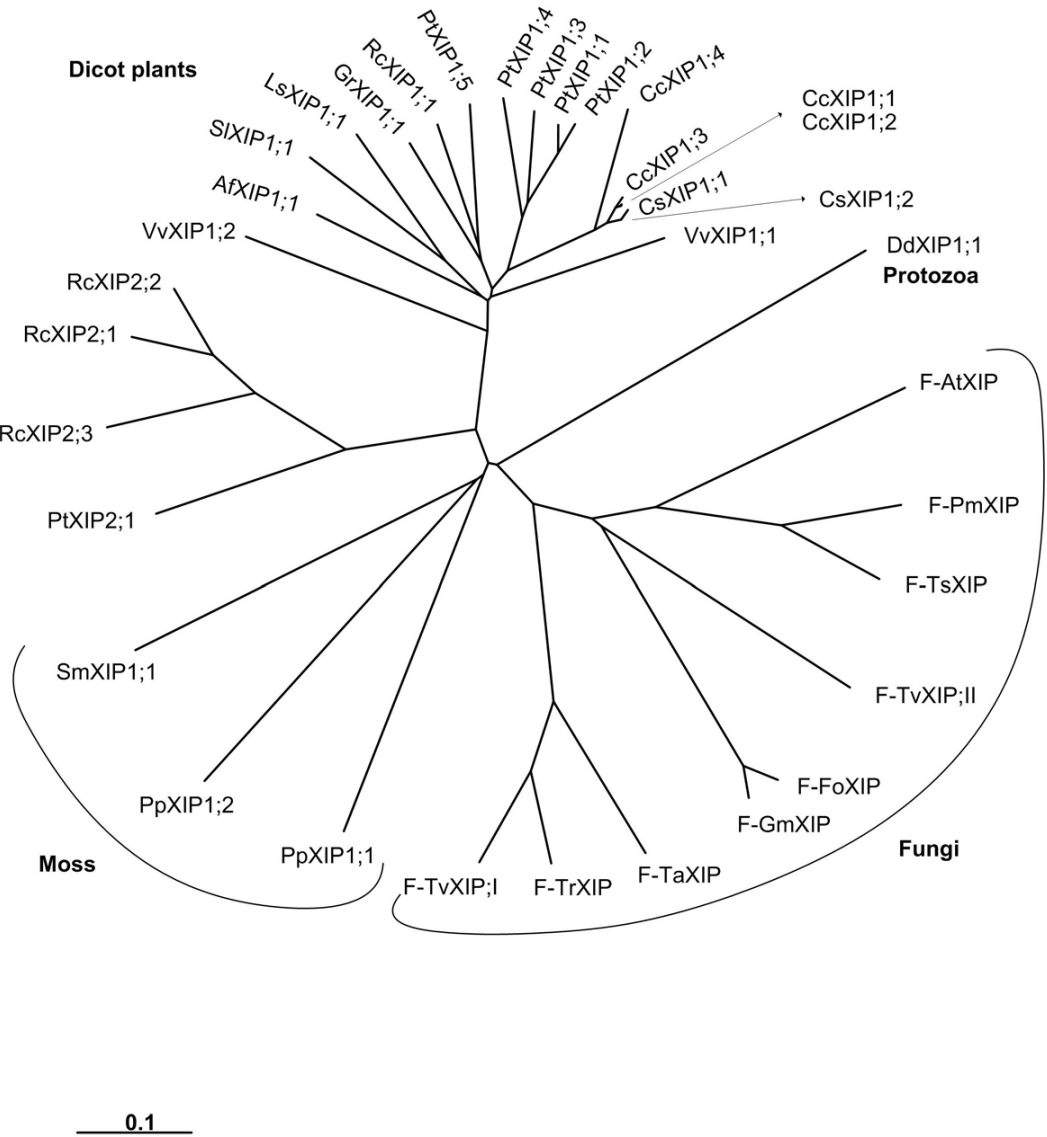
**Phylogenetic analysis of XIPs**. All 35 XIPs from dicot plants, fungi, moss and protozoa have been used to construct the phylogenetic tree using NJ method. Multiple sequence alignment for creating the phylogenetic tree was generated by T-COFFEE. XIPs from dicot plants, fungi and moss cluster separately. All dicot XIPs cluster into two subgroups XIP1 and XIP2. All fungi XIPs and some dicot XIPs (Table 2) have been identified in this study. Other dicot XIPs, moss XIPs and the lone XIP from protozoa were identified by Danielson and Johanson. The names of *Populus *XIPs, dicot XIPs identified in this study and fungi XIPs are given in Tables 1, 2 and 3 respectively. The names of other dicot and the protozoan XIPs are given as follows with their IDs (EST/RefSeq/whole genome shotgun sequence) in brackets: SlXIP1;1 -- *Solanum lycopersicum *(BT014197), CcXIP1;1 -- *Citrus clementina *(DY275505), GrXIP1;1 -- *Gossypium raimondi *(CO092422), RcXIP1;1 -- *Ricinus communis *(EG656577), RcXIP2;1 -- *Ricinus communis *(EG666650), AfXIP1;1 -- *Aquilegia formosa × Aquilegia pubescens *(DR936893 and DT742029), DdXIP1;1 -- *Dictyostelium discoideum *(XM_639170) and VvXIP1;1 and VvXIP1;2 -- *Vitis vinifera *(AM455454).

We have carried out phylogenetic analysis of all PtMIPs along with *all *XIPs. The XIPs from fungi, other dicot plants and moss clustered together with PtXIPs and are grouped separately from other *Populus *subfamilies namely PtPIPs, PtTIPs, PtNIPs and PtSIPs (Additional file 5). When only XIP members are considered, the fungi and moss XIPs form two independent clusters separate from the dicot XIPs (Figure 2). All the dicot XIPs fall into one of the two subgroups, XIP1 or XIP2. The lone XIP from protozoa does not fall into any of the four groups. Analysis of pairwise sequence alignments indicates that the XIP sequences within the subgroup are highly similar. The average sequence identities between pairs of sequences within XIP1 and XIP2 groups are ~71% and ~70% respectively (Table 4). However, the sequence variation between the two XIP groups is significant and the average sequence identity falls to ~40% when sequences are compared across the two groups. XIPs of dicot plants have diverged from those sequences from fungi and moss. The range of average sequence identities between plant XIPs and fungi/moss XIPs varies from ~27% to 34%. Among the fungi XIPs, some pairs of sequences are very similar. For example, XIP sequences from *Fusarium oxysporum *and *Gibberella moniliformis *have ~94% sequence identity. When all 36 possible fungi XIP pairs from 9 sequences are considered, the average pairwise sequence identity is only ~47%. However, there are four pairs within fungi XIPs whose sequence identity exceeds 70%. When PtXIPs are compared with other MIP subfamilies in *Populus*, namely PtPIPs, PtNIPs, PtTIPs and PtSIPs, the average pairwise sequence identities vary from 25 to 32%. This indicates that PtXIPs have diverged significantly from other subfamilies. Notably, substitutions are observed in the conserved NPA motif in loop B in almost all XIPs. However, the recent crystal structure of an MIP homolog from *Plasmodium falciparum *[58] in which both the NPA motifs are substituted, indicates that the mutations in the conserved in NPA motif are compensated by covariant mutations throughout the protein.

**Table 4:** Average pairwise sequence identities (in percentage) of XIP subfamilies from different organism groups

	Dicot XIP1s (18)^a^	Dicot XIP2s (4)^a^	Moss XIPs (3)^a^	Fungi XIPs (9)^a^	Protozoa (1)^a^
Dicot XIP1s	70.6				

Dicot XIP2s	39.9	69.5			

mossXIPs	34.3	32.0	33.9		

Fungi XIPs	32.0	27.1	30.1	47.2	

Protozoa	32.3	29.1	27.8	27.8	

^a^The number of sequences under each group is given in brackets.

### Comparison of ar/R selectivity filters in PtMIPs and XIPs

Knowledge of three-dimensional structure helps to understand the mechanism of a protein's function at molecular level. To date, the structure of only one plant MIP protein (SoPIP2;1) has been determined experimentally at atomic level [49]. Homology modeling technique has been used to build three-dimensional models of plant MIPs and it helped to identify different structural subclasses based on the residues in the ar/R selectivity filter [21,66]. Such an approach also helped to identify the group conservation of small/weakly polar residues at the helix-helix interface. We have modeled all the PtMIP proteins and the additional XIPs found in other dicot plants, fungi, moss and protozoa. We have analyzed the ar/R selectivity filters of all PtMIPs with a specific focus to XIP proteins. The non-XIP proteins from *Populus *have been compared with those from XIPs. Our structure-based sequence alignments of PtMIPs and XIPs help us to identify features in XIP proteins that distinguish them from MIPs from other subfamily.

**Figure 3:**
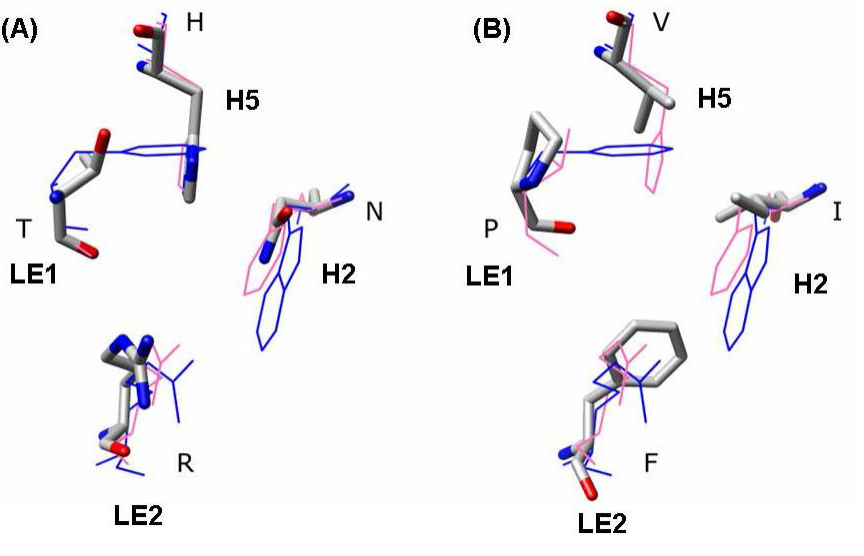
**Ar/R selectivity filters of PtPIP2;10 and PtSIP1;1**. Ar/R selectivity filters of non-XIP *Populus *MIPs that are not found in their counterparts from *Arabidopsis*, rice and maize. Transmembrane regions of the MIP models from *Populus *were superposed on the experimentally determined structures of glycerol transporter GlpF (PDB ID: 1FX8) and the water-transporting spinach aquaporin SoPIP2;1 (PDB ID: 1Z98). *Populus *MIP residues are shown in stick representation with nitrogen and oxygen atoms in blue and red colors respectively. PtMIP residues are displayed in one letter code and their corresponding positions in the selectivity filter are indicated. For comparison purpose, the ar/R filters of GlpF and SoPIP2;1 are also shown in blue and pink respectively. (A) Out of 54 PIPs from four plants, PtPIP2;10 is the only PIP in which the Phe at H2 position is substituted by an Asn, making it more hydrophilic. (B) Residues forming the ar/R filter of PtSIP1;1 are very hydrophobic. Even the normally conserved Arg at LE2 position is substituted by a hydrophobic Phe. This will be one of the most hydrophobic constrictions in known MIPs.

Analysis of ar/R selectivity filters in PtPIPs, PtTIPs, PtNIPs and PtSIPs indicate that residues forming the selectivity filter region are very similar to their counterparts in other three plants compared in this study. Only three out of 49 non-XIPs show some distinct features in this region (Table 5). With the lone exception of PtPIP2;10, all PIPs from *Arabidopsis*, rice and maize and 14 out of 15 PtPIPs have Phe from helix H2, His from helix H5, Thr and Arg from loop E (LE1 and LE2 positions) forming the ar/R selectivity region. PtPIP2;10 has Asn in the place of Phe in H2 position making the pore constriction more hydrophilic (Figure 3A). Among the PtNIPs, PtNIP1;5 is somewhat similar to the other members of PtNIP1 subgroup. However, it has two small residues in positions H5 and LE1 making the size of the constriction at this point relatively larger. Similarly, PtSIP1;1 has a unique substitution in the ar/R tetrad in which the conserved Arg in loop E is replaced by bulky hydrophobic Phe. With the other three positions occupied by hydrophobic residues (Ile in H2, Val in H5 and Pro in LE1), this could be one of the most hydrophobic pore constriction in the MIP members (Figure 3B). Overall our results suggest that majority of PtMIPs that are not XIPs have ar/R signatures similar to the ones present in *Arabidopsis*, rice and maize. This might be an indication that these MIPs from *Populus *facilitate the transport of same or similar solute molecules that are transported in other plants. In other words, only a couple of PtMIPs belonging to the four well known subfamilies could be involved in the transport of novel solute molecules that may be considered unique to *Populus*.

**Table 5:** Ar/R selectivity filters of *Populus *PIP, TIP, NIP and SIP members

MIP Members^a^	H2	H5	LE1	LE2
**PIP family**				
PtPIP1;1 to PtPIP1;5 PtPIP2;1 to PtPIP2;9	F	H	T	R
*PtPIP2;10*	*N*	*H*	*T*	*R*

**TIP family**				
PtTIP1;1 to PtTIP1;8	H	I	A	V
PtTIP2;1 to PtTIP2;4	H	I	G	R
PtTIP3;1, PtTIP3;2, PtTIP4;1	H	I	A	R
PtTIP5;1, PtTIP5;2	N	V	G	C

**NIP family**				
PtNIP1;1 to PtNIP1;4	W	V	A	R
*PtNIP1;5*	*W*	*A*	*A*	*R*
PtNIP2;1	G	S	G	R
PtNIP3;1 to PtNIP3;5	S/T/A	I/V	G/A	R

**SIP family**				
*PtSIP1;1*	*I*	*V*	*P*	*F*
*PtSIP*1;2, *PtSIP*1;3, *PtSIP*1;4	V/A	V	P	N
PtSIP2;1, PtSIP2;2	T/S	H	G	S

^a^PtMIP members and the corresponding selectivity filter residues are shown in italics if the ar/R selectivity filter is not found in *Arabidopsis*, rice or maize.

**Figure 4:**
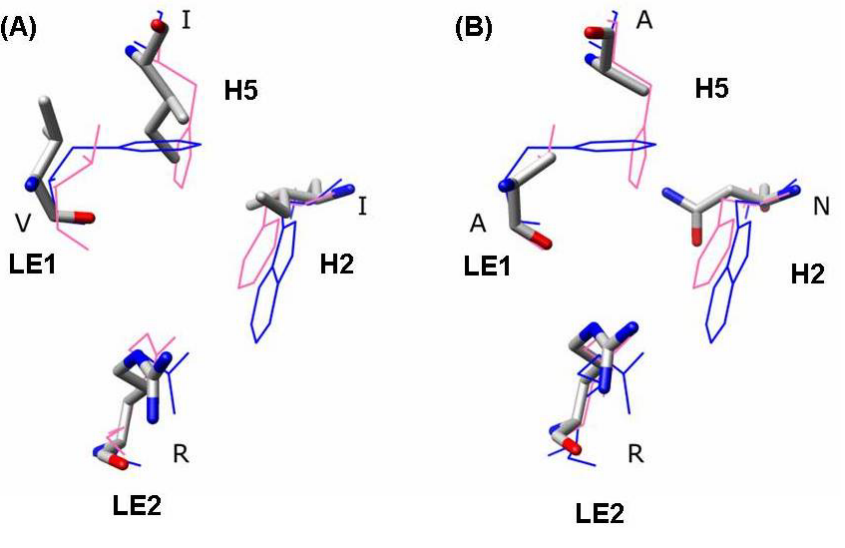
**Ar/R selectivity filters of PtXIP2;1 and F-FoXIP**. Ar/R selectivity filters of two XIPs, one from a dicot plant (PtXIP2;1) and the other from fungi (F-FoXIP). XIP models were first individually superposed on the experimentally determined structures of GlpF and SoPIP2;1 as described in Figure 3. Residues of XIP models are shown in stick representation with nitrogen and oxygen atoms displayed in blue and red respectively. For other details, see the caption of Figure 3. (A) Ar/R selectivity filter of PtXIP2;1 has three hydrophobic residues and is likely to transport a more hydrophobic solute. (B) The presence of Asn and Arg along with two small residues makes the ar/R selectivity filter of F-FoXIP more hydrophilic and result in a wider constriction. Such XIPs are likely to transport bulkier hydrophilic solutes.

Analysis of three-dimensional models of PtXIPs and other XIPs indicate that the features observed in ar/R selectivity filters are distinct in some XIPs. Among all the XIPs, dicot plant XIPs differ from those XIPs from moss and fungi. XIPs from dicots can be divided into four structural subclasses based on ar/R signatures (Table 6). In the first group, thirteen XIP sequences have Val/Ile (H2), Thr (H5), Ala (LE1) and Arg (LE2) as ar/R signature. This is similar to the ar/R filter of PtNIP3;1 and PtNIP3;2 in which the positions of hydrophobic and Thr (or Ser) residues are interchanged in the positions H2 and H5. In the second group, the Ala at LE1 position of the first group is replaced by Val making it more hydrophobic than the first group. In the third group, hydrophobic residues Val and Ile occupy three out of four positions (H2, H5 and LE1) with the conserved Arg at LE2 retained. This results in a highly hydrophobic environment at the pore constriction (Figure 4A) and it is somewhat similar to PtSIP1;1. The last group with one protein (PtXIP1;4) has ar/R tetrad similar to some of the NIP members of rice and maize (OsNIP2;1, OsNIP2;2, OsNIP3;2, OsNIP4;1, ZmNIP2;1 and ZmNIP2;2). Small residues Ala/Thr are observed in three out of four positions making the constriction larger. In general, dicot XIP members from groups II and III significantly deviate from other subfamilies of PtMIPs and display more hydrophobic character at the ar/R selectivity filter compared to other PtMIPs.

**Table 6:** Ar/R filters of 35 XIPs from different organism groups

Group^a^	XIP Members	H2	H5	LE1	LE2
	**Dicot Plants**				
I	PtXIP1;1, PtXIP1;2, PtXIP1;3 CcXIP1;1, CcXIP1;2, CcXIP1;3, CcXIP1;4 CsXIP1;1, CsXIP1;2 VvXIP1;1, VvXIP1;2 LsXIP1;1 SlXIP1;1	V/I	T	A	R
II	PtXIP1;5 GrXIP1;1 RcXIP1;1, RcXIP2;1, RcXIP2;2 AfXIP1;1	V/I	T/S	V	R
III	PtXIP2;1, RcXIP2;3	V/I	I	V	R
IV	PtXIP1;4	A	T	A	R

	**Moss**				
I	PpXIP1;1	Q	A	A	R
II	PpXIP1;2	Q	I	T	R
III	SmXIP1;1	Y	S	A	R

	**Protozoa**				
I	DdXIP	H	I	F	R

	**Fungi**				
I	F-FoXIP, F-GmXIP, F-AtXIP, F-TaXIP, F-TrXIP, F-TvXIP1, F-PmXIP	N	A/S/G	A	R/K
II	F-TsXIP	N	S	L	R
III	F-TvXIP2	A	G	F	R

^a^Structural subclasses for each organism group are defined based on the residues forming ar/R tetrad.

Comparison of ar/R filters in moss XIPs (Table 6) indicates that all three of them have different signatures and hence each one can be considered as a separate group. PpXIP1;1 has a signature similar to a TIP protein from rice and maize (OsTIP4;2 and ZmTIP4;3). Similarly, ar/R tetrad of PpXIP1;2 has resemblance to another TIP protein from rice and maize (OsTIP5;1 and ZmTIP5;1). Interestingly, these two ar/R motifs are not found in *Arabidopsis*. The third XIP from moss has a Tyr at H2 position and Tyr residue has not been observed as part of the ar/R signature in any of the 160 plant MIPs analyzed from the four plant species. The ar/R filters of all three moss XIPs are more hydrophilic than their counterparts in dicot plants.

The only example from the protozoa has bulky residues in all four positions that form the ar/R filter. Danielson and Johanson [24] have observed that this non-plant sequence from amoeba has some of the sequence characteristics such as NPA boxes and ar/R filter different from other XIPs.

Majority of fungi XIP sequences (7 out of 9 forming group I) has ar/R tetrad in which the H2 position is occupied by Asn (Table 6). Small residues are found in H5 and LE1 positions and the highly conserved Arg is observed in LE2 (F-TaXIP has a Lys residue in this position; Figure 4B). This signature is very different from that of dicot plant XIPs which are more hydrophobic. However, the group I fungi XIPs shows striking similarity with the ar/R filter of a moss XIP (PpXIP1;1) which in turn is similar to some of the rice and maize TIPs. Asn in H2 position is replaced by Gln in PpXIP1;1 and other features of ar/R filter are retained. Similarly, F-TsXIP from group II of fungi MIPs has ar/R signature similar to that of PpXIP1;2. The weakly polar and hydrophobic residues at H5 and LE1 positions are interchanged in the moss XIP. The XIP forming the third group in fungi (F-TvXIP2) is the only example that shows some similarity to group I dicot XIPs. One hydrophobic, two small/weakly polar residues with the conserved Arg at LE2 is the characteristic of ar/R motif in this group which is also shared by some members of *Populus *NIPs (PtNIP3;1 and PtNIP3;2).

In summary, PtMIPs that do not belong to XIP subfamily have ar/R selectivity filter similar to those found in *Arabidopsis*, rice and maize. Residues forming ar/R tetrad in fourteen dicot XIP sequences are found to be similar to the NIP sequences from the *Populus*, rice and maize. The ar/R selectivity filters of the remaining eight dicot XIPs are more hydrophobic in nature and lack counterparts in other subfamilies of plants considered in this study. On the other hand, the moss and fungi XIPs have ar/R constriction that are more hydrophilic and similar to rice and maize TIPs. The analysis of ar/R selectivity filters based on homology modeling shows clear distinction between dicot XIPs and moss/fungi XIPs.

### Comparison of loops in XIPs and other MIP subfamily members

Although transmembrane segments in aquaporin give structural scaffold and define the channel environment, loops connecting the TM helices also have significant role in the function of the channel such as gating [49] and could possibly be involved in selectivity also [58,81]. Among the five loops (A to E), the high conservation of residues observed in loops B and E are due to these loops possessing the NPA signature motif and their residues defining the channel interior and selectivity filter. The loop A, connecting H1 and H2, was used to discriminate the groups within *Populus *PIP family [73]. Loops C and D have been implicated in solute selectivity [58] and gating [49] respectively. Hence features observed in these loops could be an important factor in giving rise to (i) different MIP subgroups, (ii) determining the nature of solute that is transported and (iii) functioning of the channel itself. We specifically focused on the loops C and D to find out whether they could be used to discriminate PtXIPs from the other *Populus *subfamily members. We also analyzed dicot XIPs and fungi/moss XIPs separately. We first used structure-based sequence alignment to segregate sequences in the loop regions and then used T-COFFEE [79] method to align only the part belonging to the respective loop regions from all MIP sequences and also independently from the subfamilies.

#### Loop C

**Figure 5:**
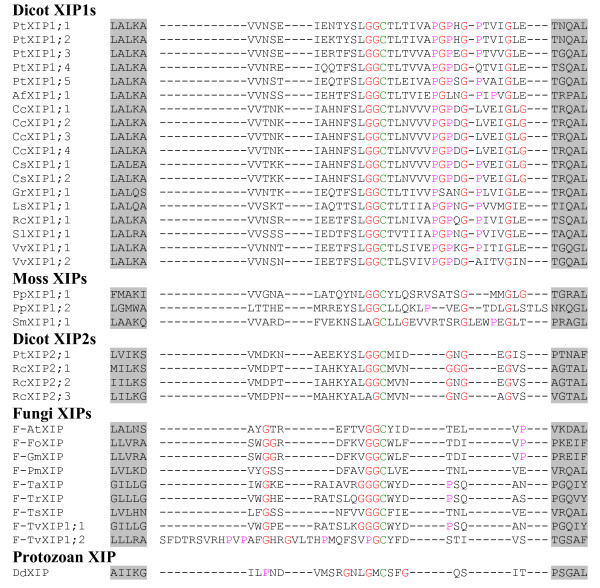
**Alignment of loop C residues of XIPs**. The sequence regions containing loop C are aligned for all XIPs. Residues forming the last turn of H3 and the first turn of H4 are shown in gray background. All Gly and Pro residues are displayed in red and pink color respectively. The conserved Cys which is part of the 'GGC' motif is shown in green.

Among the four known plant MIP subfamilies, the lengths of loop C in PIPs and a subgroup of SIPs (SIP2s) are the largest (> 20 residues) and the smallest (14 residues) respectively (Table 7). Exceptions are observed in few members. For example, ZmTIP5;1 has 29 residues. However, the same analysis for XIP members show some interesting features. In general, the length of loop C can be used to distinguish the dicot and moss XIPs from other plant MIP subfamilies. All 18 dicot XIPs belonging to the first subgroup (XIP1s) are observed to have much longer C loop with 33 residues (Figure 5). The length of the same loop in XIP2 members is shorter by 8 residues, but still 5 residues longer than plant PIPs. Surprisingly, the loop C of all moss XIPs are similar to the dicot XIP1s and all are having loop C with more than 30 residues. Fungi XIPs, on the other hand, has much shorter loop C among all XIPs and its length is comparable to that of plant PIPs with 20 residues (Figure 5).

**Table 7:** Analysis of loops C and D in MIPs from different organism groups

MIP Subfamily	No. of sequences	Length of the loop C	Loops with ≥ 3 Gly	Length of loop D	No. of basic residues in loop D
Dicot XIP1s	18	33	All dicot, XIPs	15 -- 16	≥ 3 (19 out of 22)
Dicot XIP2s	4	25			

Moss XIPs	3	31 -- 34	All	12-16	1 (2 out of 3)

Fungi XIPs	9	20 -- 23	All	16	2 (8 out of 9)

All PIPs	54	21 -- 22	53 out of 54	13 -- 14	4 (53 out of 54)

All TIPs	49	17	40 out of 49	9 -- 11	2 (41 out of 49)

All NIPs	37	16 -- 24	12 out of 37	8 -- 9	1 (32 out of 37)

All SIPs	14	14-19	None	8 -- 9	2 (10 out of 14)

Human glycerol transporting AQPs^a^	4	35	All	10	0 (2 out of 4) 2 (2 out of 4)

Other Human AQPs^b^	9	20-23	AQP1, AQP4	5 -- 9	2 basic (7 out of 9)

^a^AQP3, AQP7, AQP9, AQP10 are the human AQP homologs that transport glycerol.

^b^AQP0, AQP1, AQP2, AQP4, AQP5, AQP6, AQP8, AQP11, AQP12

Analysis of loop C residues indicates that some MIP families are enriched with Gly residues in this loop. All XIPs have at least three Gly residues and dicot XIPs have more Gly residues than any other MIPs (Table 7). Twenty out of 22 dicot XIPs have at least five Gly residues in loop C (Figure 5). Similarly, loop C in 52 out of 54 PIPs contains at least four Gly residues (Additional file 6). However, TIPs and NIPs possess less number of Gly in loop C than their counterparts in PIPs and XIPs, although some exceptions are seen. For example, OsNIP1;2 and OsNIP1;5 have respectively 9 and 7 Gly residues in loop C. SIPs have the least number of Gly (2 or 1) in this loop. The longer loop and larger number of Gly residues indicate that the loop C in dicot XIPs is much more flexible than other MIP members.

When we analyzed the loop C of human counterparts, four out of thirteen human aquaporins (AQP3, AQP7, AQP9 and AQP10) contain 35 residues in loop C and all four also possess at least 3 Gly residues (Table 7). These human MIP homologs are known to be glycerol transporters, a feature also shared by the prototype glycerol transporter, the bacterial GlpF. GlpF with 39 residues in loop C is one of the longest known in aquaporin family. Most of the other human aquaporins have loop C with 20 to 23 residues, shorter by more than 10 residues compared to their glycerol-transporting counterparts. Although, it is tempting to correlate the length of loop C with the glycerol transporting property, several plant NIPs are known to transport glycerol [65] and they have much shorter loop C and their length is only half of what is observed in dicot XIPs and GlpF. However, the fact that the loop C residues have a role to play in the selectivity of solute transport has support from experimental studies (see Discussion).

#### Loop D

**Figure 6:**
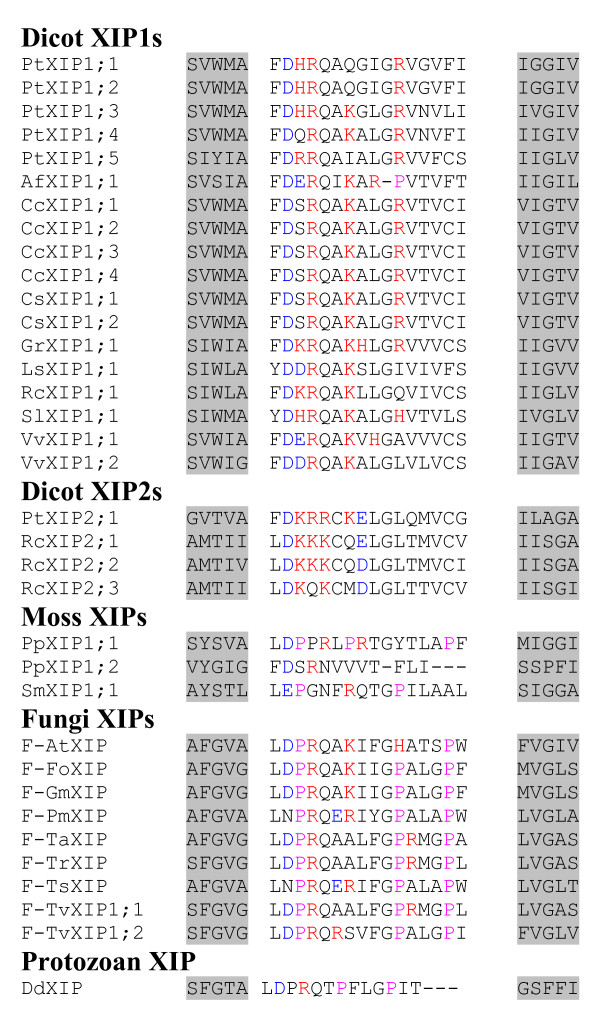
**Alignment of loop D residues of XIPs**. Multiple sequence alignment of residues forming loop D is shown for all XIPs. Residues forming the last turn of H4 and the first turn of H5 are shown in gray background. All basic (Arg, Lys and His) and acidic (Asp and Glu) residues are displayed in red and blue colors respectively. ClustalW (version 1.82) was used to perform the multiple sequence alignment of both loops.

The crystal structure of plant plasma membrane aquaporin clearly demonstrates the involvement of loop D in gating of the channel [49]. Loop D is, in general, shorter than loop C. Among the four major non-XIP subfamilies, PIPs have longer D loop with 13 to 14 residues (Additional file 7). D loops in SIPs are the shortest with 8 to 9 residues (Table 7). There are some exceptions like AtPIP1;4 and AtNIP1;1 that have more than 20 residues in loop D. Analysis of loop D sequences in XIPs indicates that all of them have slightly longer loop D (15 to 16 residues) compared to that of plant MIPs from other subfamily members (Figure 6).

Computational studies on a mammalian AQP1 have indicated that the basic residues in loop D could be significant in cation transport in the central channel formed by the tetramer [82]. We have examined the occurrence of charged residues in loop D of all plant MIP families (Table 7). In general, loop D in dicot XIPs is more basic, having at least three basic residues compared to their counterparts in moss and fungi (Figure 6). The loop D of all the fungi XIPs is rich in proline residues and no proline is observed in the same loop in majority of dicot XIPs. Among non-XIP members, PIPs have four basic residues compared to two or less in TIPs, NIPs and SIPs (Additional file 7). Similarly, two out of four glycerol-transporting human AQPs have less number of basic residues than other human homologs. This analysis indicates that the possible influence of loop D in gating of the central channel could be different in different species.

### Group conservation of residues at the helix-helix interface

Analysis of high-resolution crystal structures of MIP homologs showed that small and weakly polar residues (Ala, Gly, Ser, Thr and Cys) occur at the helix-helix interface of transmembrane helix bundle [21,54,83]. Structure-based sequence alignment of 105 MIP sequences from *Arabidopsis*, rice and maize indicated that these residues are conserved as a group at the helix-helix interface at 17 positions in MIP proteins [21]. High abundance of such residues helps to mediate helix-helix interactions and close packing of helices [84]. In this study, we have analyzed the group conservation at those 17 positions by considering 55 *Popular *MIPs and all the XIPs using structure-based sequence alignment. Our results show that in *Populus *MIPs also small and weakly polar residues are group conserved at the helix-helix interface (Table 8). As observed in the other three plant species, PtPIPs have the highest conservation in which all 17 positions are 100% group conserved (Additional file 8). This is followed by PtTIPs (82 - 100%) and PtNIPs (91 - 100%). Group conservation at helix-helix interface is in general high in PtSIPs and PtXIPs, although some positions are poorly conserved. The conservation of Ala 78, Gly 82 and Ser 181 (the numbering followed here is that of 1Z98, the structure of SoPIP2;1) is below 50% in PtSIPs. Similarly, the positions corresponding to Thr 55, Ala 103, Ser 181 and Ala 256 are either poorly conserved (< 25%) or not conserved at all in PtXIPs. It must be mentioned that the number of sequences considered for PtXIPs is only six, and analysis of all 22 dicot XIP sequences also gives rise to a similar observation (Table 8).

**Table 8:** Group conservation of small and weakly polar residues at the helix-helix interface of PtMIPs and all XIPs

Residue^a^	All PtMIPs^b, c^	Dicot XIPs^c^	Fungi XIPs^c^
T48	T(91), S, A ***(95)***	T(86), S ***(100)***	T(44), S ***(67)***
T55	G(45), T(25), S, A ***(80)***	T ***(5)***	S(33), T, A ***(67)***
A78	A(69), S, C, T ***(91)***	S(73), A ***(82)***	G(100) ***(100)***
G82	G(51), A(41) ***(91)***	A(73), S ***(77)***	---
A103	A(76), S, T ***(87)***	T, A ***(23)***	T(44), A ***(56)***
G107	G(58), A(25), S, T ***(98)***	A(50), S(32) ***(82)***	A(78), S ***(100)***
G129	G(87), A ***(100)***	G(100) ***(100)***	G(100) ***(100)***
A130	S(56), A(33), G, T ***(100)***	A(50), G(45), S ***(100)***	G(89), A ***(100)***
G133	A(55), G(45) ***(100)***	G(91), A ***(100)***	A(89), G ***(100)***
T172	T(82), G, S, A ***(100)***	T(55), S(27), A ***(100)***	S(33), T, A, C ***(89)***
S181	A(42), S(27), G, T ***(84)***	T, S ***(23)***	G(100) ***(100)***
G203	G(87), A, S ***(100)***	G(82), A ***(100)***	G(100) ***(100)***
S226	S(60), A, C, T ***(93)***	C(100) ***(100)***	C(100) ***(100)***
G248	G(73), A, C, S ***(98)***	G(95) ***(95)***	G(44), A(33), S ***(100)***
G252	G(87), A ***(98)***	A(77), S, G ***(91)***	A(56), G ***(78)***
A253	A(60), G, C, S, T ***(98)***	C(82), S, G ***(95)***	G(33), C, T, A, S ***(89)***
A256	A(71), G ***(87)***	---	---

^a^Residue numbers correspond to that of spinach aquaporin SoPIP2;1 (PDB ID: 1Z98)

^b^Group-based conservation is reported for all the five *populus *MIP subfamilies based on structure-based sequence alignment of 55 *Populus *MIPs

^c^Small and weakly polar interfacial residues in *Populus *MIP sequences and their conservation (if it exceeds 25%) are given. If the conservation is less than 25%, only the residues are given. For each position, group conservation of all the five residues (Gly, Ser, Thr, Ala and Cys) is shown in bold and italics.

Analysis of 9 fungi XIPs indicates that the group conservation of small and weakly polar residues is 100% for 9 positions and is very high for another 5 positions. There are differences between dicot and fungi XIPs. For example, at position 181, although the group conservation is only 23% in dicot XIPs, Gly is 100% conserved in fungi XIPs. However, we observed the opposite at position 82. In the dicot XIPs, the group conservation at this position is 77% while in the fungi XIPs, there is absolutely no conservation. Similarly, the position 55 is reasonably well conserved in fungi XIPs and there is poor conservation in dicot XIPs.

In the previous analysis, we have observed that subfamilies show strong preference for one or another amino acid at certain positions [21]. A similar trend is observed in *Populus *MIPs also. Notably, the position 226 is occupied by either Ser/Ala in PtPIPs, PtTIPs, PTNIPs and PtSIPs. In PtXIPs a strong preference for Cys is observed at that position (Additional file 8). Similarly, at position 253 Ala/Gly is predominantly found in the four non-XIP subfamilies and a preference for Cys is found in PtXIPs at the same position. This is also confirmed in the analysis of 35 XIPs and all of them have Cys at position 226. In position 253, only dicot XIPs shows a strong preference for Cys (Table 8).

### Gene Structure of MIPs

#### Non-XIP Populus MIPs

The availability of three plant genomes, two dicotyledons and one monocotyledon, enabled us to analyze and compare the gene structures of MIP genes belonging to different subfamilies and different species. Recently, gene structures of MIPs from the avascular plant *Physcomitrella patens *have also been analyzed [24]. Although the exon-intron organization of AtMIPs has been reported [18], comparison of MIP gene structures across the three plant species has not been carried out. We have compared the gene structures of PtMIPs with that of OsMIPs and AtMIPs. In general, they show that the number and positions of introns are unique and are conserved within each subfamily of a given species. However, major differences are observed when the subfamilies from dicots are compared with those from the monocot.

**Figure 7:**
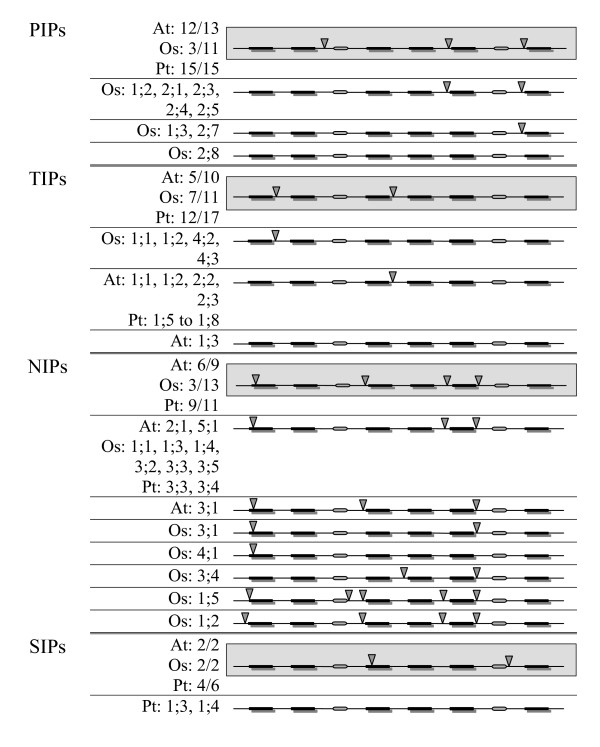
**Gene structure of non-XIP MIPs from *Populus*, *Arabidopsis *and rice**. Exon-intron organization of non-XIP MIP genes from *Populus*, *Arabidopsis *and rice is depicted for the PIP, TIP, NIP and SIP subfamilies. The exon-intron pattern observed in majority of MIPs within a subfamily is shown in gray background. In this case, only the number of MIPs having that pattern is indicated for each plant species. For example, "At:12/13" indicates that 12 out of 13 AtPIPs have the same gene structure. For those members with different exon-intron organization, the MIP name is explicitly given (example: Os:2.8 in PIP subfamily). The six TM regions are shown in black bars and the loops B and E are shown in oval shape. The intron positions are indicated by inverted triangle.

Comparison of members from PIP subfamily shows that the gene structures of majority of PtPIPs have three introns, similar to that of AtPIPs (Figure 7). However, only 3 out of 11 OsPIPs have the same organization. Eight OsPIPs have lost at least one intron (two of the OsPIPs belonging to the indica-cultivar group have been excluded from this analysis). OsPIP1;3 and OsPIP2;7 have only one intron and OsPIP2;8 has no intron. In most of the OsPIPs, the intron between the helices H2 and H3 has been lost. A similar result is observed for NIP subfamily (Figure 7). Most of the PtNIPs have gene structures similar to that of AtNIPs. Four introns are observed in 9 out 11 PtNIPs. Gene structures of OsNIPs diverged from their counterparts in *Arabidopsis *and *Populus*. At least one intron is lost in nine out of 13 OsNIPs and they have three introns or less. In most of them, the intron between the TM helices H2 and H3 is lost as in OsPIPs. Members of SIP subfamily have two introns in *Arabidopsis*, rice and also most of the *Populus *SIPs. PtSIP1;3 and PtSIP1;4 have no introns. Most of the *Populus *(12 out of 17) and rice (7 out of 11) TIP members and half of AtTIPs (5 out of 10) have two introns. Four TIP members, each from rice, *Arabidopsis *and *Populus *have lost one intron. However, it must be pointed out that the intron lost in rice TIPs is not the same as that observed in the other two dicot plant TIPs.

The gene structures of the non-XIP MIPs in two dicot plants, *Populus *and *Arabidopsis*, are very similar (Figure 7). Both PIP and NIP subfamilies have three and four introns respectively in these two plants. The number and locations of introns in the PIP and NIP subfamilies of moss plant *Physcomitrella patens *[24] are the same as that observed in their counterparts in the two dicot plants. On the other hand, intron loss is observed in rice PIP and NIP subfamilies. This could be a general feature observed in dicot and monocot PIP and NIP subfamilies and such intron loss could have happened when monocots diverged from dicots.

#### Populus XIPs versus moss/fungi XIPs

**Figure 8:**
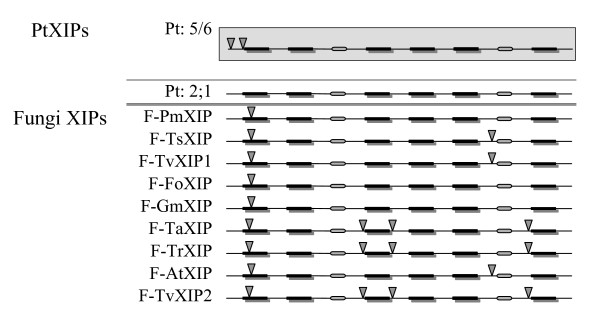
**Exon-intron pattern observed in *Populus *and fungi XIPs**. Exon-intron organization of XIP MIP genes from *Populus *and fungi is displayed. Five out of 6 PtXIPs have similar exon-intron organization and the pattern is shown in gray background. The sixth PtXIP (PtXIP2;1) has no introns and its gene structure is shown separately. The six TM regions are shown in black bars and the loops B and E are shown in oval shape. The intron positions are indicated by inverted triangle. For details about fungi XIPs, see Table 3.

The pattern of exon -introns in five out of six PtXIPs has already been reported and compared with that of two moss XIPs [24]. Two introns in the N-terminal region are observed in six out of seven XIPs. Due to the high degree of variation observed in the N-termini, no conclusion was reached regarding the conservation of intron positions between the moss plant and *Populus*. Since the fungi XIPs have been identified from their respective genome sequences, it is possible to derive the gene structure of these MIP sequences and compare them with that of *Populus *and *Physcomitrella*. It is interesting to note that in addition to the N-terminal intron, six out of nine fungi XIPs have at least one additional intron (Figure 8). In all six of them, an intron is present between helices H5 and H6. In three cases, additional introns are present between helices H2 and H3 and also between H3 and H4.

### Transcript abundance of non-PtXIPs and PtXIPs

**Figure 9:**
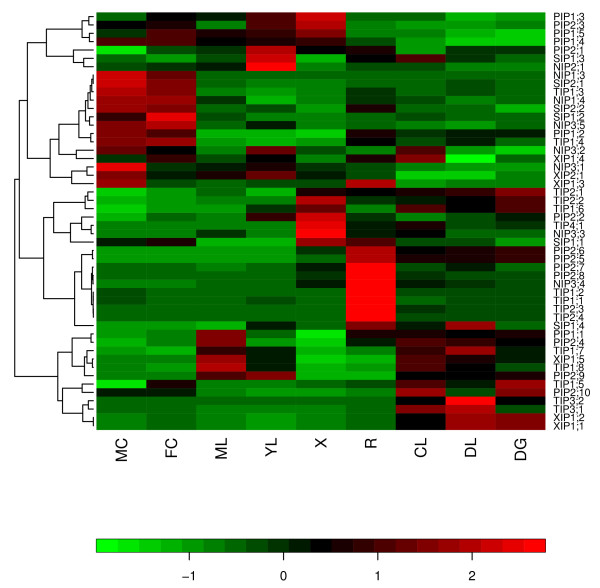
**Relative transcript abundance profiles of *Populus *MIPs**. A heat map showing the transcript abundance of MIPs from all *Populus *subfamilies [85] is displayed using the program "Heatplus" [86]. This heat map is produced using the expression data obtained from *Populus *eFP browser [103]. The transcript abundance levels for the *Populus *MIPs were clustered using hierarchical clustering based on Pearson correlation coefficients. Each row corresponds to the normalized expression profile of a particular gene and their names are shown. Data obtained for nine different tissues for each gene are represented in columns. Symbols in the map represent as follows: ML -- mature leaf; YL -- young leaf; R -- root; DG -- dark-grown seedlings, etiolated; DL -- dark grown seedling etiolated and then exposed to light for 3 hrs; CL -- continuous light-grown seedling; FC -- female catkins; MC -- male catkins; X -- xylem. The data is normalized for each gene (row-normalized). The relative transcript accumulation is represented in a color code with green and red showing respectively the lower and higher levels of transcript accumulation.

Expression levels of all *Populus *MIPs were analyzed using an Affymetrix microarray-based Poplar genome arrays [85] as described in the Methods section. We have reanalyzed the Populus transcript abundance data generated by Wilkins et al [85]. Transcript abundance of 50 out of 55 PtMIPs are available in the microarray dataset. There were no probe sets for two TIPs (PtTIP5;1 and PtTIP5;2) and three NIPs (PtNIP1;1, PtNIP1;2 and PtNIP1;5). Heatmap (Figure 9) is produced for the remaining *Populus *MIPs using the expression profiles obtained for nine different tissues (seedlings grown under three different light conditions, young and mature leaves, female and male catkins, roots and xylem). Probe sets were clustered using hierarchical clustering and the heatmap is displayed using this clustering based on the transcript abundance pattern using the program Heatplus [86]. Major PtMIPs that are expressed in xylem, a tissue responsible for the woody stem, are PtPIPs and PtTIPs. A similar result is observed in root tissues also. Maximum number of PtTIPs is expressed in seeds grown in different light conditions. PtNIPs and PtSIPs are the predominant members expressed in male and female catkins. No appreciable accumulation of transcripts in mature leaf and seedlings grown in continuous darkness is found for NIPs and SIPs. The same is true for PIPs in female catkins and seedlings grown in continuous darkness and then transferred to light for 3 hrs. PtXIPs are expressed in seven of the nine tissues studied. Only in xylem and female catkins, no member of XIPs is found to be expressed. Transcript abundance of two XIPs is found in male catkins, root and three tissues of seedlings grown in different light conditions. A single XIP is expressed in mature leaf (PtXIP1;5) and young leaf (PtXIP2;1).

## Discussion

Due to whole-genome duplication events, the number of protein-coding genes in *Populus *is more than that observed in *Arabidopsis *[67]. In the present study, we have found 55 *Populus *MIP genes and this is much higher compared to the total of 35 *Arabidopsis *MIPs. Our studies show that *Populus *has ~1.6 times MIP genes than those found in *Arabidopsis*. This agrees with the reported observation, based on comparative genomics studies, that for each *Arabidopsis *gene, 1.4 to 1.6 putative *Populus *homologs are found [67]. The number of MIPs from rice and maize is also found to be less than forty [19-21]. MIPs from these four plants have been compared. Phylogenetic analysis reveals that *Populus *MIPs can be divided into five subfamilies. The four known subfamilies PIPs, TIPs, NIPs and SIPs are present in all the four plant species considered in this study. Members of the fifth subfamily, XIPs, are uncharacterized and are absent in *Arabidopsis*, rice and maize. Recent studies have identified XIPs in the primitive plant *Physcomitrella patens*. TIPs and SIPs are present in larger number in *Populus *compared to the other three plants. While *Populus *has 17 TIPs and 6 SIPs, *Arabidopsis*, rice and maize each have only 10 to 11 TIPs and 2 to 3 SIPs. The higher number of MIPs found in *Populus *is mainly attributed to the presence of higher number of PtTIPs and PtSIPs in addition to the six PtXIPs.

### Non-XIP MIPs from eudicot genomes have similar ar/R filters and gene structures

Homology modeling was used to analyze the aromatic/arginine selectivity filters of plant MIPs. Ar/R tetrads from non-XIP PtMIPs were analyzed and compared with that of their counterparts from *Arabidopsis*, rice and maize. Ar/R filters of only three out of 49 non-XIP PtMIPs seem to be different from the other three plants. Although, the larger number of TIPs in *Populus *indicated the possible diversity in the solutes transported by this subfamily, analysis of ar/R selectivity filters of all PtTIPs indicated otherwise. They are identical to AtTIPs and no member of PtTIP was found to have ar/R filter that can be described as novel. Similarly, nine out of 11 PtNIP members have counterparts in AtNIPs. One or two examples are found in NIP and SIP members where the ar/R filter is identical or similar to rice/maize members. The analysis of ar/R selectivity filters did not find any surprises and it shows that majority of non-XIP PtMIPs are similar to their counterparts in *Arabidopsis*.

The availability of two eudicot genomes (*Populus *and *Arabidopsis*) and one monocot genome (rice) helps us to analyze and compare the gene structures of plant MIPs. Differences observed in the pattern of exon - intron organization of MIPs from these three plant species can explain the evolution of eudicot MIP gene family and also the divergence of monocot MIPs from dicots. Intron loss is observed in majority of the OsPIPs and OsNIPs compared to the same subfamilies in *Arabidopsis *and in *Populus*. The loss of introns observed in OsPIPs and OsNIPs might have occurred independently during the evolution of rice to achieve genome slimming [87]. It is also tempting to speculate that the intron loss in rice might have happened during the divergence of monocotyledonous and dicotyledonous plants that occurred about 200 million years ago (Mya) [88]. However, such generalization is possible only after analyzing plant MIP gene structures from a large number of monocot and dicot plants. In this context, we would like to point out the recent work of Roy and Penny [89] who have observed a high degree of intron loss along a wide variety of eukaryotic lineages. They have also found that intron losses have outnumbered intron gains during the evolution of plants.

### XIPs in dicots and fungi differ in Ar/R selectivity filter, loop C and gene structure

Our TBLASTN search on plant EST databases and fungi genomic sequences identified additional XIPs from dicot plants and fungi. In total, we considered 35 XIPs from dicots, fungi and moss for characterizing this new subfamily. We analyzed several features including the nature of ar/R selectivity filters, loop lengths, conservation of residues at the helix-helix interface and gene structures and these features were compared between different species groups within XIPs to understand the evolution of this uncharacterized subfamily. Comparison was also made between XIPs and other four subfamilies of *Populus*.

#### Ar/R filters in XIPs are hydrophilic in moss/fungi and more hydrophobic in dicot plants

Homology models of XIPs were analyzed and divided into structural subclasses based on the nature of residues that constitute the ar/R selectivity filters. The 22 dicot XIPs were divided into four structural subclasses. Fourteen of them from two groups are similar to the NIP subgroups from the four plants analyzed in this paper. Eight dicot XIPs from the remaining two groups have bulky hydrophobic residues occupying two/three of the four positions. Some SIPs from *Populus*, rice and maize have such arrangement although they lack the conserved Arg at LE2 position. All the three XIPs from moss and eight out of 9 fungi XIPs have hydrophilic residues occupying two out of four positions. Small residues are found in the remaining two positions of most of the fungi and all the moss XIPs. This arrangement is very similar to some of the rice and maize TIPs but it is not found in *Populus *and *Arabidopsis*. In general, the ar/R selectivity filters of fungi and moss are more hydrophilic than their dicot counterparts. This clearly indicates that the nature of solutes that are transported by dicot XIPs will be very different from their counterparts in fungi/moss.

Ar/R filters of some of the XIPs are presented in the recent work of Danielson and Johanson [24]. Two possibilities are given for the residue at H5 position in their work. In the present study, when the target sequence was aligned with the template sequences during homology modeling procedure, it resulted in aligning the conserved Gly in H5 and hence in our models, the residue at H5 position of ar/R filters is the alternate residue reported in their paper [24]. This residue is Ser/Thr in most of the cases. Even if we consider the other possibility for H5 position (Val/Ile for dicot XIPs) as reported in [24], the ar/R filter of dicot XIPs will become even more hydrophobic compared to fungi/moss XIPs. There is also a disagreement in the ar/R tetrad reported for SmXIP1;1. Our model shows that the H2 position in this moss XIP is occupied by a Tyr residue, whereas Danielson and Johanson [24] have reported a Leu residue at this position. Our structure-based sequence alignment clearly shows that Tyr is more likely to occupy this position (data not shown) which also makes the ar/R filter more hydrophilic as observed in the other two moss XIPs (PpXIP1;1 and PpXIP1;2)

#### Can the length of loop C be used as an indicator of XIP subfamilies?

Although the significance of loops B and E is one of the most well established in aquaporin channel's function, recent crystal structures from the plant spinach [49] and the malarial parasite *Plasmodium falciparum *[58] have indicated the role of two other loops D and C in the channel's gating and selectivity. Loop C connects the two halves of the channel protein linking the transmembrane segments H3 and H4. The length of this loop from known crystal structures varies from 20 residues in water-transporting human AQP1 [52] to 39 residues in glycerol-transporting GlpF [53]. This loop tucks into the channel core towards the ar/R selectivity filter and comes in close contact with the Arg residue at LE2 position of ar/R tetrad. The nature of residues in loop C is suggested to influence the solute molecules approaching the extracellular vestibule [58,81]. The length of loop C seems to be characteristic of different plant MIP subfamilies. Analysis of loop C in XIP members shows variation among the XIP subfamilies. Dicot XIP1s, moss XIPs, glycerol-specific GlpF and all four glycerol-transporting human AQP homologs have loop C that is more than 30 residues long. Dicot XIP2s and fungi XIPs have a smaller loop C with 20 to 25 residues as observed in other human AQP homlogs. Loop C in GlpF has been suggested to provide an attractive site for glycerol in the periplasmic vestibule [81]. Although, there seems to be a correlation between the nature of solute transport and the length of the loop C, this relationship could not be very clearly established. For example, it appears that all glycerol-transporting MIPs will have a long loop C with > 30 residues. However, several plant NIPs have been shown to transport glycerol [65] and some of them have much shorter loop C with less than 20 residues. Similarly, if we assume that all water-transporting channels have shorter loop C with < 25 residues, then the moss XIPs with hydrophilic ar/R selectivity filter are likely to transport water along with other hydrophilic solutes with their C loop having more than 30 residues. A clearer picture is likely to emerge if we have functional data on more MIPs that can be directly linked with the length of loop C.

We have also recognized another interesting feature that some of the MIP families are enriched with Gly residues in loop C. Dicot XIPs and PIPs have at least five and four Gly residues respectively in loop C. It could be that these Gly residues are present to impart flexibility to the loop or they could adopt conformations that are not allowed for other residues. We have examined the conformations of the three Gly residues that are part of the 'GGG' motif in both chains of spinach PIP structure (PDB ID: 1Z98; [49]). All three Gly residues have a positive φ value (+66 to +102°) and a ψ value close to zero (-12 to + 17°) and this conformation is not accessible to other residues. Hence it is possible that Gly residues in the "GGGxN" and "GGC" motifs of PIPs and XIPs in loop C could play an important conformational role.

#### Intron loss is observed in moss and Populus XIPs

Exon-intron pattern helps us to understand the evolution of XIP genes. Since all the *Populus *and fungi genes were identified from their respective genome sequences and the gene structure of *Physcomittrella *XIPs have been already reported [24], it was possible to compare the exon-intron organization of these 17 XIPs (6 from *Populus*, 9 from fungi and 2 from *P. Patens*). While six out of 9 fungi MIPs have at least two introns, a single intron at the N-terminus is found in *Populus *and the moss XIPs. It appears that intron loss has occurred during the evolution when the moss plants diverged from fungi with moss XIPs having retained only the N-terminal intron. When moss plants further diverged to dicotyledons, no introns were inserted in the coding region of XIPs. While the gene structures of moss and dicot XIPs are similar, the fungi with more introns have different pattern of exon-intron organization.

#### Subfunctionalization of XIPs: Expression profiles of non-PtXIPs vs PtXIPs

Members of *Populus *XIP family are expressed in seven out of nine tissues indicating that they don't show any tissue-specific transcript abundance. The fact that XIPs are not found in monocots and they are not particularly specific to any tissue indicates that the functions of XIP members might have been taken over by other MIP members during evolution. Clustering on the basis of transcript abundance pattern shows that PtXIP1;1 and PtXIP1;2 are grouped with PtTIP3;1 and PtTIP3;2. Analysis of ar/R selectivity filters of these members does indicate similar features. The positions of a bulky hydrophobic residue and a polar residue at H2 and H5 positions in PtXIP1;1 and PtXIP1;2 are exchanged in PtTIP3;1 and PtTIP3;2. Similarly, the transcript accumulation of PtXIP1;5 is most similar to PtTIP1;8 and both their ar/R tetrads have two bulky hydrophobic residues. PtXIP1;3 and PtXIP1;4 have closely related transcription abundance profiles with PtNIP3;1 and PtNIP3;2. Both the groups share two small residues in the ar/R selectivity filters. Thus analysis of *Populus *microarray data has indeed cast a light on likely members that could replace PtXIPs in monocots. It is possible that the XIP members became "functionally redundant" during evolution and the above TIP and NIP members could have substituted the functions of the redundant XIPs.

#### Evolution of dicot XIPs

**Figure 10:**
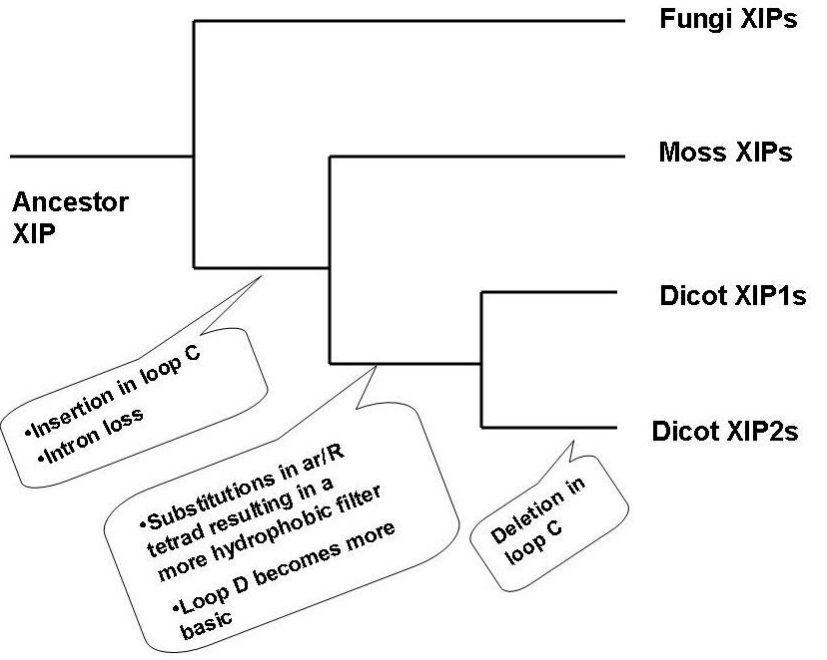
**Evolution of dicot XIPs**. A likely scenario for the evolution of dicot XIPs. The evolutionary events are indicated at the point where the XIPs diverged. Dicot XIPs evolved from fungi and moss through substitutions at ar/R selectivity filter, insertion/deletion of loop C and loss of an intron. However, small and weakly polar residues occurring at the helix-helix interface are highly group-conserved.

Several reports, including fossil studies and molecular clock estimates have speculated the animal and plant evolutionary lines. Recent protein sequence analysis has estimated that major lineages of fungi were present more than 1000 million years ago and land plants appeared after 300 million years [90]. Analysis of MIPs from primitive organisms to higher animals will help to understand the evolution of these channel proteins and their transport mechanisms of diverse solutes at molecular level. Analysis of ar/R selectivity filters, loops and exon-intron organization of 34 XIPs from fungi, moss and dicot plants has given an idea about the evolution of this subfamily of aquaporins from fungi to higher plants (XIP from protozoa is not included in this Discussion). The hydrophilic ar/R selectivity filter in the fungi and moss XIPs indicates that these MIPs are likely to be involved in transport of hydrophilic solutes including water. The emergence of higher plants could possibly indicate more diversity in the solutes that are transported. The amino acids in the hydrophilic ar/R selectivity filters of fungi and moss XIPs were substituted by hydrophobic residues during the divergence of higher plants and this selectivity filter has become more hydrophobic in the dicot XIPs. As a result, the dicot XIPs are likely to be involved in solutes that are more hydrophobic than those transported by their counterparts in fungi and moss. With no XIP homolog found in monocots, at least the XIPs might have been replaced by some of the TIPs and NIPs with similar ar/R selectivity filters and transcription abundance profiles. The loop C in moss XIPs is longer than that of fungi XIPs and hence an insertion of more than 10 residues has occurred in the loop C of moss XIPs. While this length is retained in XIP1 group of dicot plants, the dicot XIP2s have loop C that is shorter by 8 residues. Hence, a deletion event seems to have occurred when dicot XIP2s evolved from moss or diverged from dicot XIP1s. As far as the loop D is concerned, dicot XIPs have more basic residues in loop D than their counterparts in fungi/moss and the only other subfamily with more number of basic residues in loop D is PIPs. As suggested for AQP1 [82], loop D in XIPs could be involved in activating the central tetrameric ion channel upon binding to some signaling molecule. Although evolution has made its mark in the selectivity filters and loops, the group conservation of small and weakly polar residues in the helix-helix interface of the α-helical bundle observed in other MIP subfamilies is largely maintained in XIPs also. Analysis of exon-intron pattern suggests that intron loss has occurred in XIP genes when fungi diverged from the lineage leading to primitive and higher plants. In summary, during divergence from fungi and moss, the ar/R selectivity filters of dicot XIPs has become more hydrophobic, loop C has become longer in a subgroup of dicot XIPs and loop D has become more basic. Moreover, analysis of gene structure indicates that moss/*Populus *XIPs lost introns when they evolved from fungi. The evolutionary features observed for dicot XIPs are summarized in Figure 10. Some of the observations made in this study will be strengthened as more genome sequences are available for different kingdoms and we will have a better understanding of the evolution of MIPs at molecular level.

## Conclusion

We have analyzed 55 *Populus *MIP sequences and compared them with those from *Arabidopsis*, rice and maize. In addition to the four known MIP subfamilies, *Populus *has an additional uncharacterized XIP subfamily. The non-XIP *Populus *members are similar to their counterparts in the other three plants. The ar/R selectivity filters of majority of PtMIPs and the characteristics of loops C and D are similar to AtMIPs, OsMIPs and ZmMIPs. As far as the gene structures are concerned, the number and positions of introns are conserved within each subfamily of a given species. However, the inter-species comparison indicates that PIPs and NIPs of monocots lost introns when they diverged from eudicots.

We have also characterized 35 XIPs belonging to four different taxonomic groups. Our results show that in comparison to a hydrophilic selectivity filters in fungi and moss XIPs, substitutions in ar/R selectivity filters led to a more hydrophobic constriction in dicot XIPs. A longer loop C due to insertion is observed when moss and a subgroup of dicot XIPs evolved from fungi. When fungi XIPs diverged, intron loss is observed in moss and dicot XIPs. Analysis of microarray data indicates that *Populus *XIPs are expressed in almost all the tissues studied and they don't show any unique tissue-specific expression. While substitutions in ar/R tetrad and insertion/deletion events in loops reflect the divergence of these channel proteins, a high conservation of small and weakly polar residues as a group at the helix-helix interface is observed in all MIP subfamilies. Presumably, such group conservation helps to maintain the structural integrity of this channel protein during evolution. Our results indicate that in comparison to their counterparts in fungi and moss, dicot XIPs are likely to transport more hydrophobic solutes. Loop C in dicot XIPs in general and XIP1 subgroup in particular will have a potential influence in the selectivity of the solutes.

## Methods

### Identification of *Populus *MIP genes

The genome sequence of *Populus trichocarpa *female individual "Nisqually 1" clone [67] was searched for MIP genes using TBLASTN [76,80]. The whole genome shotgun sequence (WGS) of *Populus *available at the National Center for Biotechnology Information (NCBI) [75] was used for this purpose with a rice MIP protein sequence (OsPIP2;1) as a query sequence. The hits thus obtained were subjected to phylogenetic clustering (see below). One representative sequence from each cluster was chosen as query sequence to identify additional and more distantly related *Populus *MIP homologs. Regions in *Populus *WGS contigs containing MIP genes were used to find out the gene structure using the program GeneMark.hmm ES-3.0 [77,78]. This version of GeneMark program is based on self-training algorithm for prediction of genes from novel eukaryotic genomes. There is significant similarity between *Populus *and *Arabidopsis *at the genome level and also the relative frequency of protein domains [67]. Between these two organisms, there is similarity in the codon usage also [70]. Hence, for gene prediction in *Populus*, *Arabidopsis *was chosen as a model organism in GeneMark. The predicted MIP genes were further compared with the *Populus *EST sequences available at NCBI and also the *Populus *EST database "PopulusDB" [70,72]. The KOG (euKaryotic Orthologous Groups) browser in Joint Genome Institute (JGI) [91] was also looked for *Populus *MIP genes.

The program T-COFFEE [79] was used to perform multiple sequence alignment on MIP protein sequences which was then used to generate phyologenetic tree. Three different methods were used to construct the evolutionary relationship among the sequences. They include neighbor-joining method as implemented in Clustal (Version 1.82) [92] and heuristic searches using distance and parsimony methods as available in PAUP* version 4.0.0d55 in GCG package (Wisconsin Package version 10.3, Accelrys Inc., San Diego, California). The stability of branches in the resulting trees was confirmed by 100 bootstrap trails for all the three methods. The program TreeView [93] was used to display the trees.

### Homology modeling of plant MIPs

Three-dimensional models of *Populus *MIPs and other MIP proteins were built using the same protocol described in our earlier studies [21] to build models of *Arabidopsis*, rice and maize MIPs and it is briefly described below. Modeling procedure consisted of two stages. In the first stage, the software package MODELLER [94,95] was used to construct homology models of plant MIPs. In the second stage, the program SCWRL3 [96] was used to predict the side-chain conformation. MODELLER derives a set of spatial restraints on the structure of the target sequence using its alignment with the sequence of template structure(s). The resulting model is derived by optimizing the violations of all spatial restraints. The quality of the model is usually improved by considering more than one structure as template. In this study, we used the structures of mammalian AQP1 [52], bacterial GlpF [53] and archael AQPM [55] simultaneously as templates in their comparative modeling procedure. Their unique PDB IDs are 1J4N, 1FX8 and 2F2B respectively. All are high resolution structures (resolution 1.7 to 2.2 Å) and show different water permeabilities [96]. Using the program 'GAP' available in GCG package and the scoring matrix BLOSUM62, we found the pairwise sequence alignment of all *Populus *MIPs with the three template sequences. The average pairwise sequence identities between *Populus *MIPs and the three templates range from 21 to 45%. Template sequences were first aligned based on a multiple structural superposition and then the target sequence was aligned. The target-template alignment was manually checked to find out if there is any gap in the middle of a transmembrane helical region or in the conserved loops B or E. If necessary, this alignment was manually refined. We have also analyzed more than 800 MIP sequences from diverse organisms (Gupta and Sankararamakrishnan, Manuscript in preparation) and found that at least one residue in each transmembrane segment (E17, G59, Q103, E144, G175 and P218 respectively in TM1 to TM6; 1J4N numbering) is very highly conserved. We have exploited this information during the alignment of target and template sequences and hence there is less ambiguity in transmembrane segments. The models were built with the resultant target-template alignment using a 'very fast' simulated annealing optimization protocol. Ten models were built for each target sequence and the one with the lowest MODELLER objective function was selected. The refinement of loops and the side-chain conformations of non-conserved residues were carried out by MODELLER's loop optimization procedure and the graph theory-based SCWRL3 [96] method respectively. Finally, the model was minimized using GROMACS [97,98] and its stereochemical quality was evaluated using PROCHECK [99]. Pore diameter profile of the model along its pore axis was calculated using the program HOLE [100] as described in Bansal and Sankararamakrishnan [21].

#### *Populus *microarray analysis

The transcript abundance of all *Populus *MIPs was analyzed using PopGenExpress, an Affymetrix microarray-based resource for poplar transcriptome analysis [85]. Expression data was obtained in biological triplicate RNA samples extracted in nine tissues by Malcolm Campbell and coworkers [85] and we have reanalyzed this transcript abundance data to find out whether there is any pattern of transcript accumulation in *Populus *MIP members. The microarray data corresponding to these experiments can be accessed in the NCBI's GEO database [101] (accession number: GES13990). Probe sets corresponding to the putative *Populus *MIPs were identified using Probe Match, a tool available as part of the NetAffx Analysis Center [102]. The identified probe sets were then used in the *Populus *electronic fluorescent pictograph browser (Poplar eFP browser) [103] to find out the transcript abundance levels. For genes with more than one probe sets, the median of expression values were considered. When two genes have the same probe set, then they are considered to have same level of transcript accumulation. The probe sets were then clustered using hierarchical clustering based on Pearson coefficients and the program Heatplus available in Bioconductor package [86] was used to display the expression pattern.

## Authors' contributions

RS conceived the project. RS and ABG designed the work. ABG carried out the work. RS and ABG wrote the manuscript. Both authors approved the final version of the manuscript.

## Supplementary Material

Additional file 1 **Discarded MIP sequences from JGI list and in fungi genomic analysis**. Nine *Populus *sequences from JGI (Table S1) and 5 fungi MIP sequences (Table S2) were excluded from the analysis of MIP sequences. Their JGI/NCBI accession codes and the reasons for discarding these sequences are given. Click here for file

Additional file 2 **Phylogenetic tree constructed for *Populus *and rice MIPs**. Phylogenetic analysis of all *Populus *MIPs is shown along with MIPs from rice. Neighbor-Joining (NJ) method was used to create this unrooted tree and NJ method used the multiple sequence alignment generated by T-COFFEE to generate the tree. *Populus *MIP subfamilies PtPIPs, PtTIPs, PtNIPs and PtSIPs clustered with the corresponding rice MIP subfamilies. XIPs observed only in *Populus *clustered separately. Each MIP subfamily is shown with a specific background color to distinguish them from others. A similar result is obtained when the same analysis was carried out with *Arabidopsis *and maize MIPs (Figure 1, Additional files 3 and 4). Click here for file

Additional file 3 **Phylogenetic tree constructed for *Populus *and maize MIPs**. Phylogenetic analysis of all *Populus *MIPs is shown along with MIPs from maize. Neighbor-Joining (NJ) method was used to create this unrooted tree and NJ method used the multiple sequence alignment generated by T-COFFEE to generate the tree. *Populus *MIP subfamilies PtPIPs, PtTIPs, PtNIPs and PtSIPs clustered with the corresponding maize MIP subfamilies. XIPs observed only in *Populus *clustered separately. Each MIP subfamily is shown with a specific background color to distinguish them from others. A similar result is obtained when the same analysis was carried out with *Arabidopsis *and rice MIPs (Figure 1, Additional files 2 and 4). Click here for file

Additional file 4 **Phylogenetic tree constructed for all four plant MIPs**. Phylogenetic analysis of all *Populus *MIPs is shown along with MIPs from the other three plants *Arabidopsis*, rice and maize. Neighbor-Joining (NJ) method was used to create this rooted tree and NJ method used the multiple sequence alignment generated by T-COFFEE to generate the tree. *Populus *MIP subfamilies PtPIPs, PtTIPs, PtNIPs and PtSIPs clustered with the corresponding MIP subfamilies from the other three plants. XIPs observed only in *Populus *clustered separately. Each MIP subfamily is marked separately. A similar result is obtained when the same analysis was carried out on *Populus *MIPs paired individually with *Arabidopsis*, rice and maize MIPs (Figure 1, Additional files 2 and 3). Numbers above branches indicate bootstrap support. *Populus *aquaporins belonging to different subfamilies are shown in different colors. Click here for file

Additional file 5 **Phylogenetic analysis of *Populus *MIPs and all XIPs**. Phylogenetic analysis of all *Populus *MIPs is shown along with XIPs from the other dicot plants, fungi, moss and protozoa. Neighbor-Joining (NJ) method was used to create this rooted tree and NJ method used the multiple sequence alignment generated by T-COFFEE to generate the tree. Members of *Populus *XIP subfamily clustered with other XIPs and this group is separate from PtPIPs, PtTIPs, PtNIPs and PtSIPs. Background of non-XIP subfamilies are shown in different colors to distinguish from each other and from XIPs. Click here for file

Additional file 6 **Sequence alignment of loop C residues of all non-XIP plant MIPs**. The sequence regions containing loop C are aligned for all non-XIP plant MIPs from *Arabidopsis*, *Populus*, rice and maize. Residues forming the last turn of H3 and the first turn of H4 are shown in gray background. All Gly and Pro residues are displayed in red and pink color respectively. The conserved residues within each subgroup are shown in green color. Click here for file

Additional file 7 **Sequence alignment of loop D residues of all non-XIP plant MIPs**. Multiple sequence alignment of loop D residues of all non-XIP plant MIPs. Residues forming the last turn of H4 and first turn of H5 are displayed in gray background. Acidic (Asp and Glu) and basic (Arg, Lys and His) residues are shown in blue and red respectively. Click here for file

Additional file 8 **Group conservation of small and weakly polar conserved interfacial residues in *Populus *MIPs**. Conservation of small and weakly polar residues (Gly, Ala, Ser, Thr, Cys) as a group is reported for all *Populus *MIPs and also individually for the subfamily members. Conservation was found out for 17 positions using structure-based sequence alignment of MIP sequences. The residues at these 17 positions occur at the helix-helix interface of the transmembrane helix bundle. Click here for file

## References

[B1] Zhou Y Setz N Niemietz C Qu H Offler CE Tyerman SD Patrick JW Aquaporins and unloading of phloem-imported water in coats of developing bean seeds Plant Cell Environ 2007 30 1566 1577 10.1111/j.1365-3040.2007.01732.x 17927694

[B2] Siefritz F Tyree MT Lovisolo C Schubert A Kaldenhoff R PIP1 plasma membrane aquaporins in tobacco: From cellular effects to function in plants Plant Cell 2002 14 869 876 10.1105/tpc.000901 11971141 PMC150688

[B3] Maurel C Plant aquaporins: Novel functions and regulation properties FEBS Lett 2007 581 2227 2236 10.1016/j.febslet.2007.03.021 17382935

[B4] Bots M Vergeldt F Wolters-Arts M Weterings K van As H Mariani C Aquaporins of the PIP2 class are required for efficient anther dehiscence in tobacco Plant Physiol 2005 137 1049 1056 10.1104/pp.104.056408 15734911 PMC1065405

[B5] Kaldenhoff R Fischer M Functional aquaporin diversity in plants Biochim Biophys Acta 2006 1758 1134 1141 10.1016/j.bbamem.2006.03.012 16730645

[B6] Higuchi T Suga S Tsuchiya T Hisada H Morishima S Okada Y Maeshima M Molecular cloning, water channel activity and tissue sepcific expression of two isoforms of radish vacuolar aquaporin Plant Cell Physiol 1998 39 905 913 9816675 10.1093/oxfordjournals.pcp.a029453

[B7] Kjellbom P Larsson C Johansson I Karlsson M Johanson U Aquaporin and water homeostasis in plants Trends Plant Sci 1999 4 308 314 10.1016/S1360-1385(99)01438-7 10431220

[B8] Gao YP Young L Bonham-Smith P Gusta LV Characterization and expression of plasma and tonoplast membrane aquaporins in primed seed of Brassica napus during germination under stress conditions Plant Mol Biol 1999 40 635 644 10.1023/A:1006212216876 10480387

[B9] Kaldenhoff R Ribas-Carbo M Sans JF Lovisolo C Heckwolf M Uehlein N Aquaporins and plant water balance Plant Cell Environ 2008 31 658 666 10.1111/j.1365-3040.2008.01792.x 18266903

[B10] Uehlein N Kaldenhoff R Aquaporins and plant leaf movements Annals Botany 2008 101 1 4 10.1093/aob/mcm278 18024416 PMC2701841

[B11] Lian HL Yu X Ye Q Ding XS Kitagawa Y Kwak SS Su WA Tang ZC The role of aquaporin RWC3 in drought avoidance in rice Plant Cell Physiol 2004 45 481 489 10.1093/pcp/pch058 15111723

[B12] Peng YH Lin WL Cai WM Arora R Overexpression of a Panax ginseng tonoplast aquaporin alters salt tolerance, drought tolerance and cold acclimation ability in transgenic Arabidopsis plants Planta 2007 226 729 740 10.1007/s00425-007-0520-4 17443343

[B13] Katsuhara M Koshio K Shibasaka M Hayashi Y Hayakawa T Kasamo K Over-expression of a barley aquaporin increased the shoot/root ratio and raised salt sensitivity in transgenic rice plants Plant Cell Physiol 2003 44 1378 1383 10.1093/pcp/pcg167 14701933

[B14] Mut P Bustamante C Martinez G Alleva K Sutka M Civello M Amodeo G A fruit-specific plasma membrane aquaporin subtype PIP1;1 is regulated during strawberry (Fragaria × ananassa) fruit ripening Physiol Plantarum 2008 132 538 551 18248507 10.1111/j.1399-3054.2007.01046.x

[B15] Agre P Preston GM Smith BL Jung JS Raina S Moon C Guggino WB Nielsen S Aquaporin Chip - The archetypal molecular water channel Am J Physiol 1993 265 F463 F476 7694481 10.1152/ajprenal.1993.265.4.F463

[B16] Ishibashi K Sasaki S Fushimi K Uchida S Kuwahara M Saito H Furukawa T Nakajima K Yamaguchi Y Gojobori T Molecular cloning and expression of a member of the aquaporin family with permeability to glycerol and urea in addition to water expressed at the basolateral membrane of kidney collecting duct cells Proc Natl Acad Sci USA 1994 91 6269 6273 10.1073/pnas.91.14.6269 7517548 PMC44182

[B17] Maurel C Reizer J Schroeder JI Chrispeels MJ Saier MH Functional characterization of the *Escherichia coli *glycerol facilitator, GlpF, in *Xenopus oocytes* J Biol Chem 1994 269 11869 11872 7512955

[B18] Johanson U Karlsson M Johansson I Gustavsson S Sjovall S Fraysse L Weig AR Kjellbom P The complete set of genes encoding major intrinsic proteins in Arabidopsis provides a framework for a new nomenclature for major intrinsic proteins in plants Plant Physiol 2001 126 1358 1369 10.1104/pp.126.4.1358 11500536 PMC117137

[B19] Chaumont F Barrieu F Wojcik E Chrispeels MJ Jung R Aquaporins constitute a large and highly divergent protein family in maize Plant Physiol 2001 125 1206 1215 10.1104/pp.125.3.1206 11244102 PMC65601

[B20] Sakurai J Ishikawa F Yamaguchi T Uemura M Maeshima M Identification of 33 rice aquaporin genes and analysis of their expression and function Plant Cell Physiol 2005 46 1568 1577 10.1093/pcp/pci172 16033806

[B21] Bansal A Sankararamakrishnan R Homology modeling of major intrinsic proteins in rice, maize and Arabidopsis: comparative analysis of transmembrane helix association and aromatic/arginine selectivity filters BMC Struct Biol 2007 7 27 10.1186/1472-6807-7-27 17445256 PMC1866351

[B22] Johanson U Gustavsson S A new subfamily of major intrinsic proteins in plants Mol Biol Evol 2002 19 456 461 11919287 10.1093/oxfordjournals.molbev.a004101

[B23] Gustavsson S Lebrun A-S Norden K Chaumont F Johanson U A novel plant major intrinsic protein in Physcomitrella patens most similar to bacterial glycerol channels Plant Physiol 2005 139 287 295 10.1104/pp.105.063198 16113222 PMC1203378

[B24] Danielson JAH Johanson U Unexpected complexity of the aquaporin gene family in the moss Physcomitrella patens BMC Plant Biol 2008 8 45 10.1186/1471-2229-8-45 18430224 PMC2386804

[B25] Zelazny E Borst JW Muylaert M Batoko H Hemminga MA Chaumont F FRET imaging in living maize cells reveals that plasma membrane aquaporins interact to regulate their subcellular localization Proc Natl Acad Sci USA 2007 104 12359 12364 10.1073/pnas.0701180104 17636130 PMC1941474

[B26] Ma JF Tamai K Yamaji N Mitani N Konishi S Katsuhara M Ishiguro M Murata Y Yano M A silicon transporter in rice Nature 2006 440 688 691 10.1038/nature04590 16572174

[B27] Takano J Wada M Ludewig U Schaaf G von Wiren N Fujiwara T The Arabidopsis major intrinsic protein NIP5;1 is essential for efficient boron uptake and plant development under boron limitation Plant Cell 2006 18 1498 1509 10.1105/tpc.106.041640 16679457 PMC1475503

[B28] Liu LH Ludewig U Gassert B Frommer WB von Wiren N Urea transport by nitorgen-regulated tonoplast intrinsic proteins in Arabidopsis Plant Physiol 2003 133 1220 1228 10.1104/pp.103.027409 14576283 PMC281617

[B29] Maeshima M Ishikawa F ER membrane aquaporins in plants Pflugers Arch Eur J Physiol 2008 456 709 716 10.1007/s00424-007-0363-7 17924135

[B30] Maurel C Verdoucq L Luu D-T Santoni V Plant aquaporins: Membrane channels with multiple integrated functions Annu Rev Plant Biol 2008 59 595 624 10.1146/annurev.arplant.59.032607.092734 18444909

[B31] Maurel C Aquaporins and water permeability of plant membranes Annu Rev Plant Physiol Plant Mol Biol 1997 48 399 429 10.1146/annurev.arplant.48.1.399 15012269

[B32] Dean RM Rivers RL Zeidel ML Roberts DM Purification and functional reconstitution of soybean nodulin 26. An aquaporin with water and glycerol transport properties Biochemistry 1999 38 347 353 10.1021/bi982110c 9890916

[B33] Biela A Grote K Otto B Hoth S Hedrich R Kaldenhoff R The Nicotiana tabacum plasma membrane aquaporin NtAQP1 is mercury-insensitive and permeable for glycerol Plant J 1999 18 565 570 10.1046/j.1365-313X.1999.00474.x 10417707

[B34] Wu B Beitz E Aquaporins with selectivity for unconventional permeants Cell Mol Life Sci 2007 64 2413 2421 10.1007/s00018-007-7163-2 17571212 PMC11138416

[B35] Gaspar M Bousser A Sissoeff I Roche O Hoarau J Mahe A Cloning and characterization of ZmPIP1-5b, an aquaporin transporting water and urea Plant Sci 2003 165 21 31 10.1016/S0168-9452(03)00117-1

[B36] Kojima S Bohner A von Wiren N Molecular mechanisms of urea transport in plants J Memb Biol 2006 212 83 91 10.1007/s00232-006-0868-6 17264988

[B37] Gerbeau P Guclu J Ripoche P Maurel C Aquaporin Nt-TIPa can account for the high permeability of tobacco cell vacuolar membrane to small neutral solutes Plant J 1999 18 577 587 10.1046/j.1365-313x.1999.00481.x 10417709

[B38] Choi WG Roberts DM Arabidopsis NIP2;1, a major intrinsic protein transporter of lactic acid induced by anoxic stress J Biol Chem 2007 282 24209 24218 10.1074/jbc.M700982200 17584741

[B39] Dordas C Brown PH Evidence for channel mediated transport of boric acid in squash (Cucurbita pepo) Plant and Soil 2001 235 95 103 10.1023/A:1011837903688

[B40] Bienert GP Schussler MD Jahn TP Metalloids: essential, beneficial or toxic? Major intrinsic proteins sort it out Trends Biochem Sci 2008 33 20 26 10.1016/j.tibs.2007.10.004 18068370

[B41] Bienert GP Thorsen M Schuessler MD Nilsson HR Wagner A Tamas MJ Jahn TP A subgroup of plant aquaporins facilitate the bidirectional diffusion of As(OH)3 and Sb(OH)3 across membranes BMC Biol 2008 6 26 10.1186/1741-7007-6-26 18544156 PMC2442057

[B42] Katsuhara M Hanba YT Barley plasma membrane intrinsic proteins (PIP aquaporins) as water and CO2 transporters Pflugers Arch Eur J Physiol 2008 456 687 691 10.1007/s00424-007-0434-9 18330597

[B43] Bienert GP Moller ALB Kristiansen KA Schulz A Moller IM Schjoerring JK Jahn TP Specific aquaporins facilitate the diffusion of hydrogen peroxide across membranes J Biol Chem 2007 282 1183 1192 10.1074/jbc.M603761200 17105724

[B44] Bertl A Kaldenhoff R Function of a separate NH_3_-pore in aquaporin TIP2;2 from wheat FEBS Lett 2007 581 5413 5417 10.1016/j.febslet.2007.10.034 17967420

[B45] Jahn TP Moller ALB Zeuthen T Holm LM Klaerke DA Mohshin B Kuhlbrandt W Schjoerring JK Aquaporin homologues in plants and mammals transport ammonia FEBS Lett 2004 574 31 36 10.1016/j.febslet.2004.08.004 15358535

[B46] Santoni V Verdoucq L Sommerer N Vinh J Pflieger D Maurel C Methylation of aquaporins in plant plasma membrane Biochem J 2006 400 189 197 10.1042/BJ20060569 16839310 PMC1635436

[B47] Wallace IS Choi WG Roberts DM The structure, function and regulation of the nodulin 26-like protein family of plant aquaglyceroporins Biochim Biophys Acta 2006 1758 1165 1175 10.1016/j.bbamem.2006.03.024 16716251

[B48] Daniels MJ Yeager M Phosphorylation of aquaporin PvTIP3;1 defined by mass sepctrometry and molecular modeling Biochemistry 2005 44 14443 14454 10.1021/bi050565d 16262244

[B49] Tornroth-Horsefield S Wang Y Hedfalk K Johanson U Karlsson M Tajkhorshid E Neutze R Kjellbom P Structural mechanism of plant aquaporin gating Nature 2006 439 688 694 10.1038/nature04316 16340961

[B50] Vera-Estrella R Barkla BJ Bohnert HJ Pantoja O Novel regulation of aquaporins during osmatic stress Plant Physiol 2004 135 2318 2329 10.1104/pp.104.044891 15299122 PMC520800

[B51] Prak S Hem S Boudet J Viennois G Sommerer N Rossignol M Maurel C Santoni V Multiple phosphorylations in the C-terminal tail of plant plasma membrane aquaporins Mol Cell Proteomics 2008 7 1019 1030 10.1074/mcp.M700566-MCP200 18234664

[B52] Sui H Han B-G Lee JK Walian P Jap BK Structural basis of water-specific transport through the AQP1 water channel Nature 2001 414 872 878 10.1038/414872a 11780053

[B53] Fu D Libson A Miercke LJW Weitzman C Nollert P Krucinski J Stroud RM Structure of a glycerol-conducting channel and the basis for its selectivity Science 2000 290 481 486 10.1126/science.290.5491.481 11039922

[B54] Savage DF Egea PF Robles-Colmenares Y O'Connell JD III Stroud RM Architecture and selectivity in aquaporins: 2.5 A structure of aquaporin Z PLoS Biol 2003 1 334 340 10.1371/journal.pbio.0000072 PMC300682 14691544

[B55] Lee JK Kozono D Remis J Kitagawa Y Agre P Stroud RM Structural basis for conductance by the archaeal aquaporin AqpM at 1.68 A Proc Natl Acad Sci USA 2005 102 18932 18937 10.1073/pnas.0509469102 16361443 PMC1323191

[B56] Gonen T Sliz P Kistler J Cheng Y Walz T Aquaporin-0 membrane junctions reveal the structure of a closed water pore Nature 2004 429 193 197 10.1038/nature02503 15141214

[B57] Harries WEC Akhavan D Miercke LJW Khademi S Stroud RM The channel architecture of aquaporin 0 at a 2.2-A resolution Proc Natl Acad Sci USA 2004 101 14045 14050 10.1073/pnas.0405274101 15377788 PMC521118

[B58] Newby ZER O'Connell JD III Robles-Colmenares Y Khademi S Miercke LJW Stroud RM Crystal structure of the aquaglyceroporin PfAQP from the malarial parasite *Plasmodium falciparum* Nature Struct Mol Biol 2008 15 619 625 10.1038/nsmb.1431 PMC2568999 18500352

[B59] Hub JS de Groot BL Does CO2 permeate through aquaporin-1? Biophys J 2006 91 842 848 10.1529/biophysj.106.081406 16698771 PMC1563782

[B60] de Groot BL Grubmuller H The dynamics and energetics of water permeation and proton exclusion in aquaporins Curr Opin Struct Biol 2005 15 176 183 10.1016/j.sbi.2005.02.003 15837176

[B61] de Groot BL Grubmuller H Water permeation across biological membranes: Mechanism and dynamics of aquaporin-1 and GlpF Science 2001 294 2353 2357 10.1126/science.1062459 11743202

[B62] Tajkhorshid E Nollert P Jensen MO Miercke LJW O'Connell JD III Stroud RM Schulten K Control of the selectivity of the aquaporin water channel family by global orientational tuning Science 2002 296 525 530 10.1126/science.1067778 11964478

[B63] Jensen MO Park S Tajkhorshid E Schulten K Energetics of glycerol conduction through aquaglyceroporin GlpF Proc Natl Acad Sci USA 2002 99 6731 6736 10.1073/pnas.102649299 11997475 PMC124471

[B64] Beitz E Wu B Holm LM Schultz JE Zeuthen T Point mutations in the aromatic/arginine region in aquaporin 1 allow passage of urea, glycerol, ammonia and protons Proc Natl Acad Sci USA 2006 103 269 274 10.1073/pnas.0507225103 16407156 PMC1326162

[B65] Wallace IS Roberts DM Distinct transport selectivity of two structural subclasses of the nodulin-like intrinsic protein family of plant aquaglyceroporin channels Biochemistry 2005 44 16826 16834 10.1021/bi0511888 16363796

[B66] Wallace IS Roberts DM Homology modeling of representative subfamilies of Arabidopsis major intrinsic proteins: Classification based on the Aromatic/Arginine selectivity filter Plant Physiol 2004 135 1059 1068 10.1104/pp.103.033415 15181215 PMC514140

[B67] Tuskan GA DiFazio S Jansson S Bohlmann J Grigoriev I Hellsten U Putnam N Ralph S Rombauts S Salamov A The genome of black cottonwood, *Populus trichocarpa *(Torr. & Gray) Science 2006 313 1596 1604 10.1126/science.1128691 16973872

[B68] Jansson S Douglas CJ *Populus *: A model system for plant biology Annu Rev Plant Biol 2007 58 435 458 10.1146/annurev.arplant.58.032806.103956 17280524

[B69] Brunner AM Busov VB Strauss SH Poplar genome sequence: functional genomics in an ecologically dominant plant species Trends Plant Sci 2004 9 49 56 10.1016/j.tplants.2003.11.006 14729219

[B70] Sterky F Bhalerao RR Unneberg P Segerman B Nilsson P Brunner AM Charbonnel-Campaa L Lindvall JJ Tandre K Strauss SH A Populus EST resource for plant functional genomics Proc Natl Acad Sci USA 2004 101 13951 13956 10.1073/pnas.0401641101 15353603 PMC518859

[B71] Sjodin A Bylesjo M Skogstrom O Eriksson D Nilsson P Ryden P Jansson S Karlsson J UPSC-BASE - Populus transcriptomics online Plant J 2006 48 806 817 10.1111/j.1365-313X.2006.02920.x 17092314

[B72] PopulusDB http://www.populus.db.umu.se/

[B73] Marjanovic Z Uehlein N Kaldenhoff R Zwiazek JJ Weib M Hampp R Nehls U Aquaporins in poplar: What a difference symbiont makes Planta 2005 222 258 268 10.1007/s00425-005-1539-z 15883833

[B74] Kohler A Delaruelle C Martin D Encelot N Martin F The poplar root transcriptome: analysis of 7000 expressed sequence tags FEBS Lett 2003 542 37 41 10.1016/S0014-5793(03)00334-X 12729894

[B75] NCBI http://www.ncbi.nlm.nih.gov/

[B76] TBLASTN http://www.ncbi.nlm.nih.gov/genome/seq/BlastGen/BlastGen.cgi?taxid=3694

[B77] Lomsadze A Ter-Hovhannisyan V Chernoff Y Borodovsky M Gene identification in novel eukaryotic genomes by self-training algorithm Nucleic Acids Res 2005 33 6494 6506 10.1093/nar/gki937 16314312 PMC1298918

[B78] GeneMark http://exon.gatech.edu/GeneMark/

[B79] Notredame C Higgins DG Heringa J T-Coffee: A novel method for fast and accurate multiple sequence alignment J Mol Biol 2000 302 205 217 10.1006/jmbi.2000.4042 10964570

[B80] Altschul SF Madden TL Schaffer AA Zhang J Zhang Z Miller W Lipman DJ Gapped BLAST and PSI-BLAST: a new generation of protein database search programs Nucleic Acids Res 1997 25 3389 3402 10.1093/nar/25.17.3389 9254694 PMC146917

[B81] Wang Y Schulten K Tajkhorshid E What makes an aquaporin channel a glycerol channel? A comparative study of AqpZ and GlpF Structure 2005 13 1107 1118 10.1016/j.str.2005.05.005 16084383

[B82] Yu J Yool AJ Schulten K Tajkhorshid E Mechanism of gating and ion conductivity of a possible tetrameric pore in aquaporin-1 Structure 2006 14 1411 1423 10.1016/j.str.2006.07.006 16962972

[B83] Senes A Ubarretxena-Belandia I Engelman DM The Cα-H...O hydrogen bond: A determinant of stability and specificity in transmembrane helix interactions Proc Natl Acad Sci USA 2001 98 9056 9061 10.1073/pnas.161280798 11481472 PMC55372

[B84] Eilers M Patel AB Liu W Smith SO Comparison of helix interactions in membrane and soluble α-bundle proteins Biophys J 2002 82 2720 2736 10.1016/S0006-3495(02)75613-0 11964258 PMC1302060

[B85] Wilkins O Nahal H Foong J Provart NJ Campbell MM Expansion and diversification of the Populus R2R3-MYB family of transcription factors Plant Physiol 2009 149 981 993 10.1104/pp.108.132795 19091872 PMC2633813

[B86] Heatplus http://www.bioconductor.org/packages/bioc/html/Heatplus.html

[B87] Petrov DA Lozovskaya ER Hartl DL High intrinsic rate of DNA loss in Drosophila Nature 1996 384 346 349 10.1038/384346a0 8934517

[B88] Laroche J Li P Bousquet J Mitochondrial DNA and monocot-dicot divergence time Mol Biol Evol 1995 12 1151 1156

[B89] Roy SW Penny D Patterns of intron loss and gain in plants: Intron-loss-dominated evolution and genome-wide comparison of *O. Sativa *and *A. thaliana* Mol Biol Evol 2007 24 171 181 10.1093/molbev/msl159 17065597

[B90] Heckman DS Geiser DM Eidell BR Stauffer RL Kardos NL Hedges SB Molecular evidence for the early colonization of land by fungi and plants Science 2001 293 1129 1133 10.1126/science.1061457 11498589

[B91] Joint Genome Institute http://genome.jgi-psf.org/Poptr1_1/Poptr1_1.home.html

[B92] Thompson JD Higgins DG Gibson TJ Clustal W - Improving the sensitivity of progressive multiple sequence alignment through sequence weighting, position-specific gap penalties and weight matrix choice Nucleic Acids Res 1994 22 4673 4680 10.1093/nar/22.22.4673 7984417 PMC308517

[B93] Page RDM TREEVIEW: An application to display phylogenetic trees on personal computers CABIOS 1996 12 357 358 8902363 10.1093/bioinformatics/12.4.357

[B94] Sali A Blundell TL Comparative protein modeling by satisfaction of spatial restraints J Mol Biol 1993 234 779 815 10.1006/jmbi.1993.1626 8254673

[B95] MODELLER http://www.salilab.org/modeller/

[B96] Canutescu AA Shelenkov AA RL Dunbrack Jr A graph-theory algorithm for rapid protein side-chain prediction Protein Sci 2003 12 2001 2014 10.1110/ps.03154503 12930999 PMC2323997

[B97] Lindahl E Hess B Spoel D van der GROMACS 3.0: a package for molecular simulation and trajectory analysis J Mol Modeling 2001 7 306 317

[B98] GROMACS http://www.gromacs.org/

[B99] Laskowski RA MacArthur MW Moss DS Thornton JM PROCHECK-A program to check the stereochemical quality of protein structures J Appl Cryst 1993 26 Part 2 283 291 10.1107/S0021889892009944

[B100] Smart OS Neduvelil JG Wang X Wallace BA Sansom MSP HOLE: A program for the analysis of the pore dimensions of ion channel structural models J Mol Graphics 1996 14 354 360 10.1016/S0263-7855(97)00009-X 9195488

[B101] Gene Expression Omnibus http://www.ncbi.nlm.nih.gov/geo/

[B102] NetAffx™ Analysis Center https://www.affymetrix.com/analysis/index.affx

[B103] Poplar eFP Browser http://bar.utoronto.ca/efppop/cgi-bin/efpWeb.cgi

