# Supplementary material for: Genome-wide analysis of major intrinsic proteins in the tree plant Populus trichocarpa: Characterization of XIP subfamily of aquaporins from evolutionary perspective
Source: BMC Plant Biol. 2009 Nov 20;9:134. doi: 10.1186/1471-2229-9-134 (PMC2789079; doi:10.1186/1471-2229-9-134)
Supplement: Additional file 1 — Discarded MIP sequences from JGI list and in fungi genomic analysis. Nine Populus sequences from JGI (Table S1) and 5 fungi MIP sequences (Table S2) were excluded from the analysis of MIP sequences. Their JGI/NCBI accession codes and the reasons for discarding these sequences are given. [file 1471-2229-9-134-S1.PDF]

Table S1: Discarded sequences from JGI list of *Populus* MIPs

JGI Accession	Length	Reason(s) for exclusion
562841	110	small and missing conserved loops
567478	59	small and missing conserved loops
579650	132	small (has loopE similar to those in PtXIPs)
587423	153	small and missing conserved loops
680648	196	partial sequence (identical to PtTIP2;1)
590872	234	belongs to the endophyte bacteria *Ralstonia metallidurans*
206485	224	Features do not correspond to that of aquaporin
263239	138	partial sequence
417298	73	partial sequence

Table S2: Discarded fungi MIP sequences from TBLASTN search of fungi genomic sequences

NCBI Accession code	Length in predicted sequence	Organism	Reason(s) for exclusion
AACM02000152	302	*Gibberella zeae*	Missing H2 andLB
AAIH02000095	282	*Aspergillus flavus*	Missing H5 andLE
AAJN01000140, XP_001214173	279	*Aspergillus terreus*	Missing LE andH6
AAIH02000016	290	*Aspergillus flavus*	Missing LE andH6
AAIH02000540	305	*Aspergillus flavus*	Missing LE andH6
