# Supplementary figures and images for: Genome-wide analysis of major intrinsic proteins in the tree plant Populus trichocarpa: Characterization of XIP subfamily of aquaporins from evolutionary perspective
Source: BMC Plant Biol. 2009 Nov 20;9:134. doi: 10.1186/1471-2229-9-134 (PMC2789079; doi:10.1186/1471-2229-9-134)

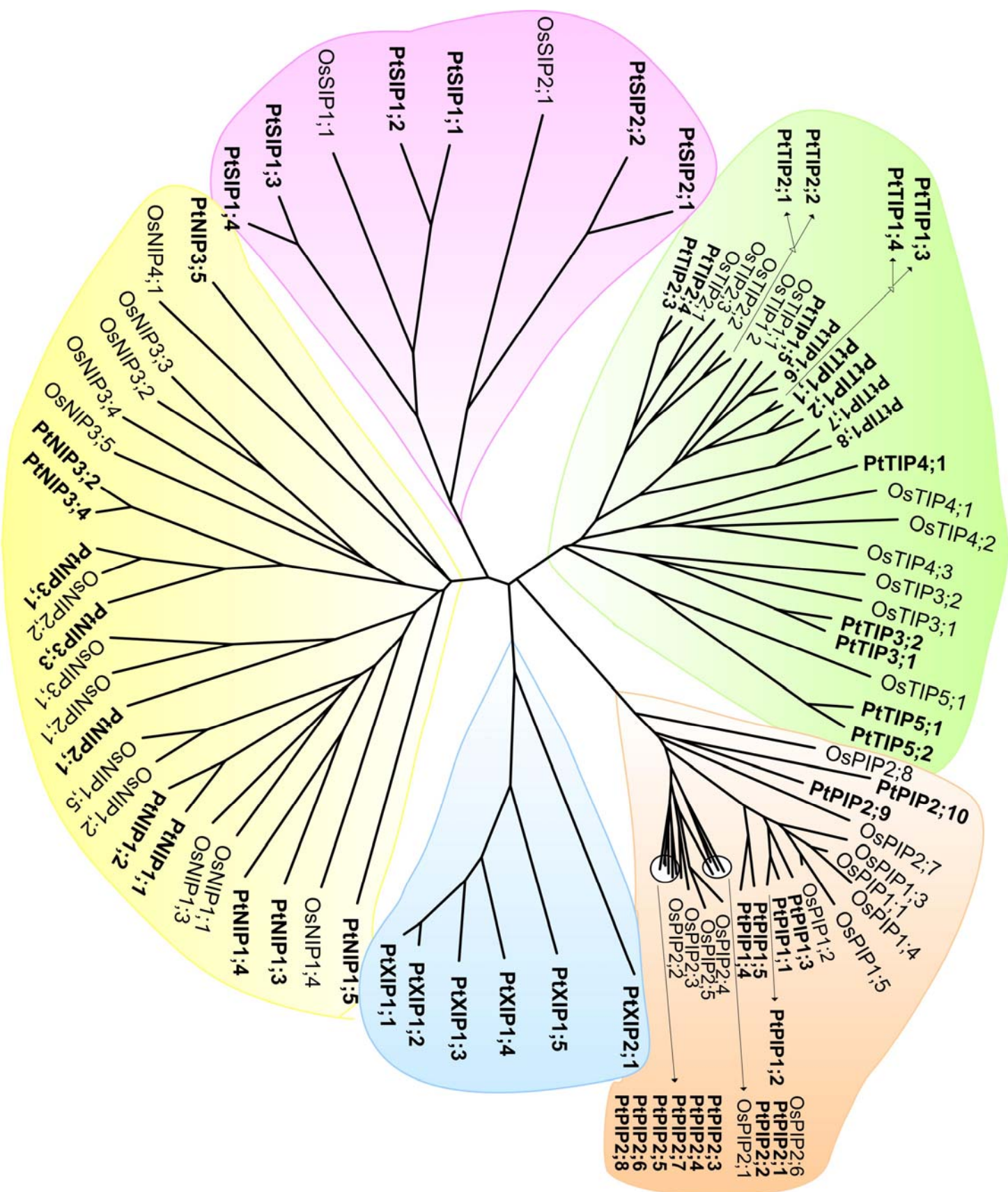

Supplement: Additional file 2 — Phylogenetic tree constructed for Populus and rice MIPs. Phylogenetic analysis of all Populus MIPs is shown along with MIPs from rice. Neighbor-Joining (NJ) method was used to create this unrooted tree and NJ method used the multiple sequence alignment generated by T-COFFEE to generate the tree. Populus MIP subfamilies PtPIPs, PtTIPs, PtNIPs and PtSIPs clustered with the corresponding rice MIP subfamilies. XIPs observed only in Populus clustered separately. Each MIP subfamily is shown with a specific background color to distinguish them from others. A similar result is obtained when the same analysis was carried out with Arabidopsis and maize MIPs (Figure 1, Additional files 3 and 4). [file 1471-2229-9-134-S2.PDF]

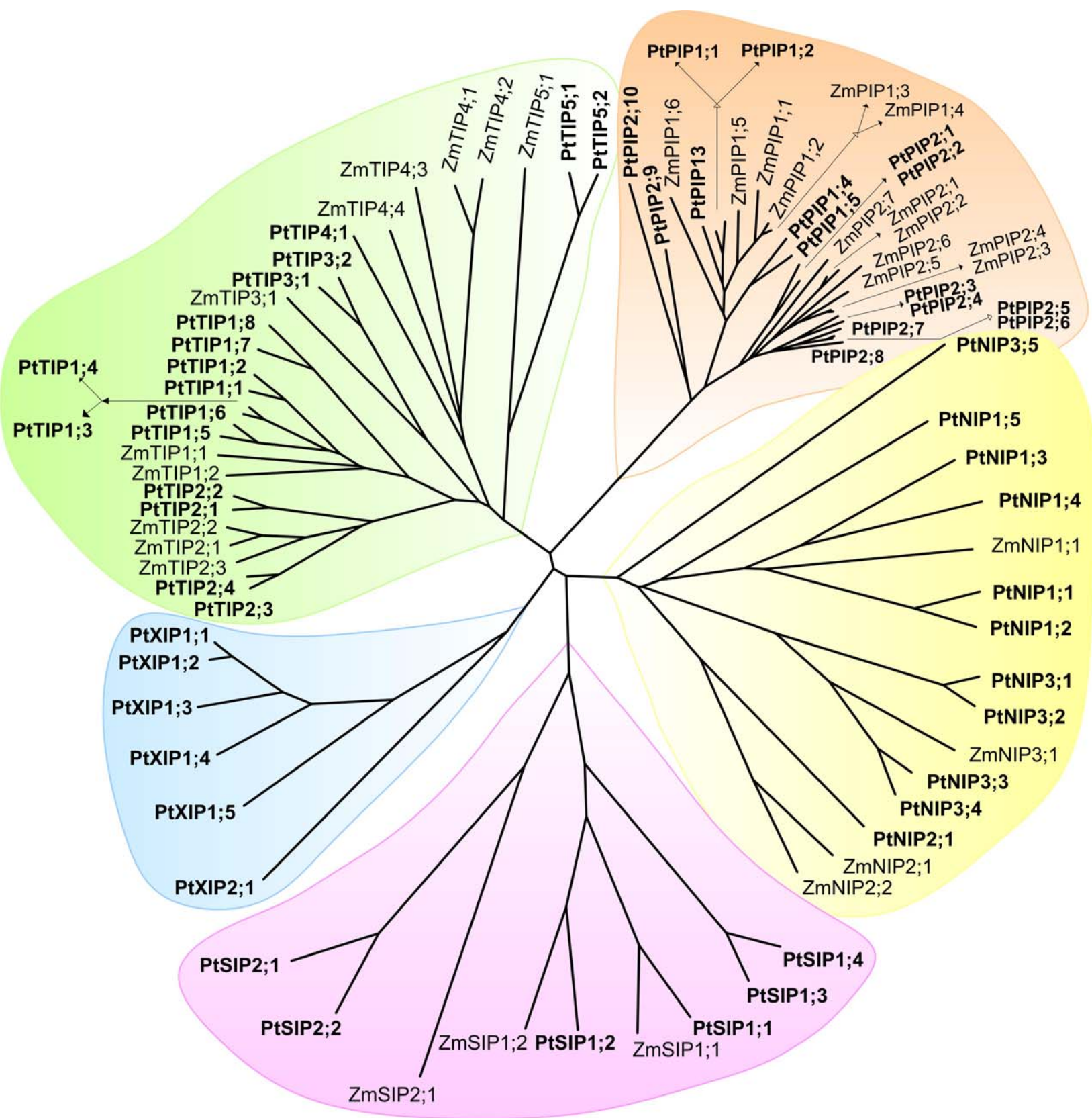

0.1

Supplement: Additional file 3 — Phylogenetic tree constructed for Populus and maize MIPs. Phylogenetic analysis of all Populus MIPs is shown along with MIPs from maize. Neighbor-Joining (NJ) method was used to create this unrooted tree and NJ method used the multiple sequence alignment generated by T-COFFEE to generate the tree. Populus MIP subfamilies PtPIPs, PtTIPs, PtNIPs and PtSIPs clustered with the corresponding maize MIP subfamilies. XIPs observed only in Populus clustered separately. Each MIP subfamily is shown with a specific background color to distinguish them from others. A similar result is obtained when the same analysis was carried out with Arabidopsis and rice MIPs (Figure 1, Additional files 2 and 4). [file 1471-2229-9-134-S3.PDF]

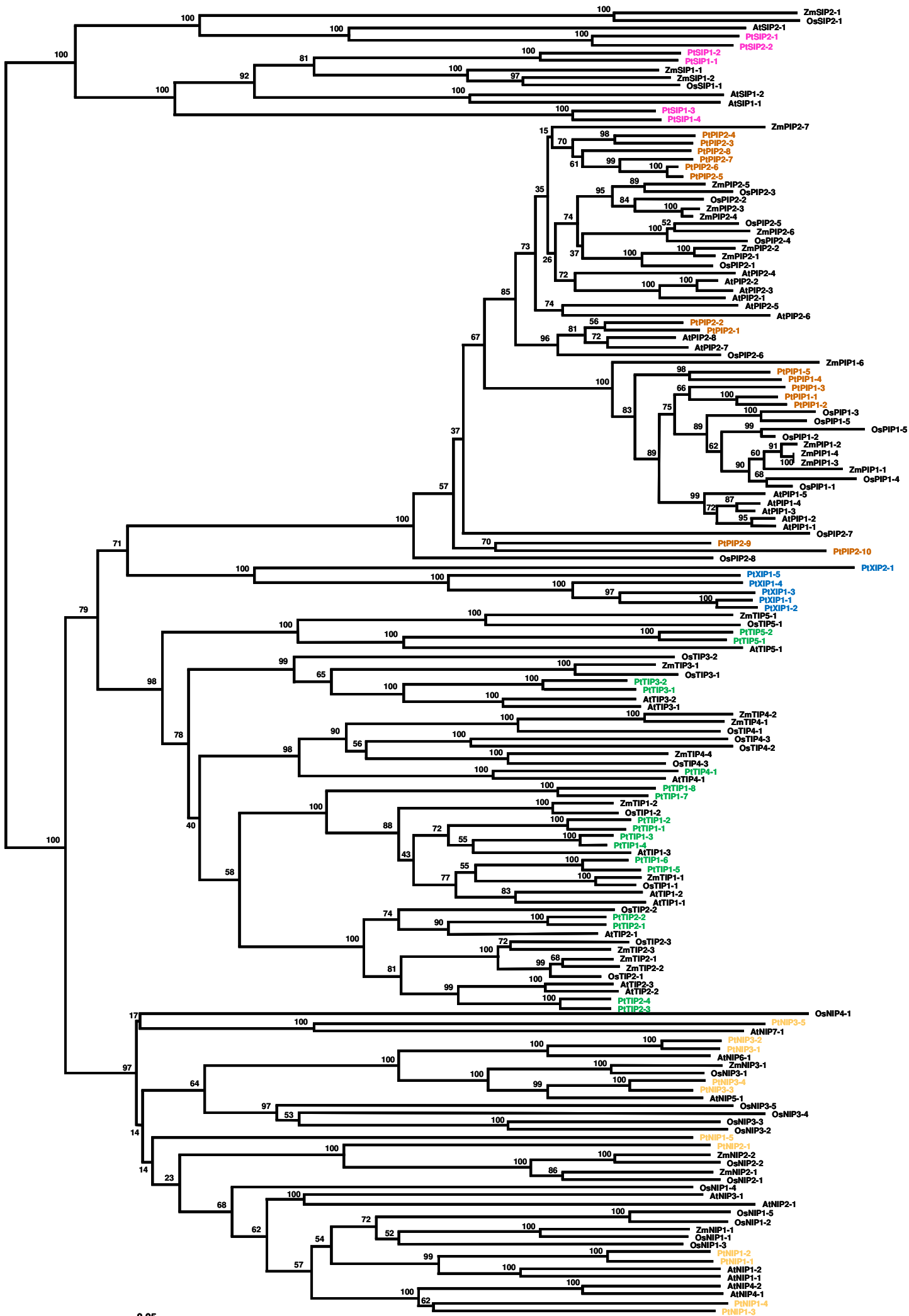

Supplement: Additional file 4 — Phylogenetic tree constructed for all four plant MIPs. Phylogenetic analysis of all Populus MIPs is shown along with MIPs from the other three plants Arabidopsis, rice and maize. Neighbor-Joining (NJ) method was used to create this rooted tree and NJ method used the multiple sequence alignment generated by T-COFFEE to generate the tree. Populus MIP subfamilies PtPIPs, PtTIPs, PtNIPs and PtSIPs clustered with the corresponding MIP subfamilies from the other three plants. XIPs observed only in Populus clustered separately. Each MIP subfamily is marked separately. A similar result is obtained when the same analysis was carried out on Populus MIPs paired individually with Arabidopsis, rice and maize MIPs (Figure 1, Additional files 2 and 3). Numbers above branches indicate bootstrap support. Populus aquaporins belonging to different subfamilies are shown in different colors. [file 1471-2229-9-134-S4.PDF]

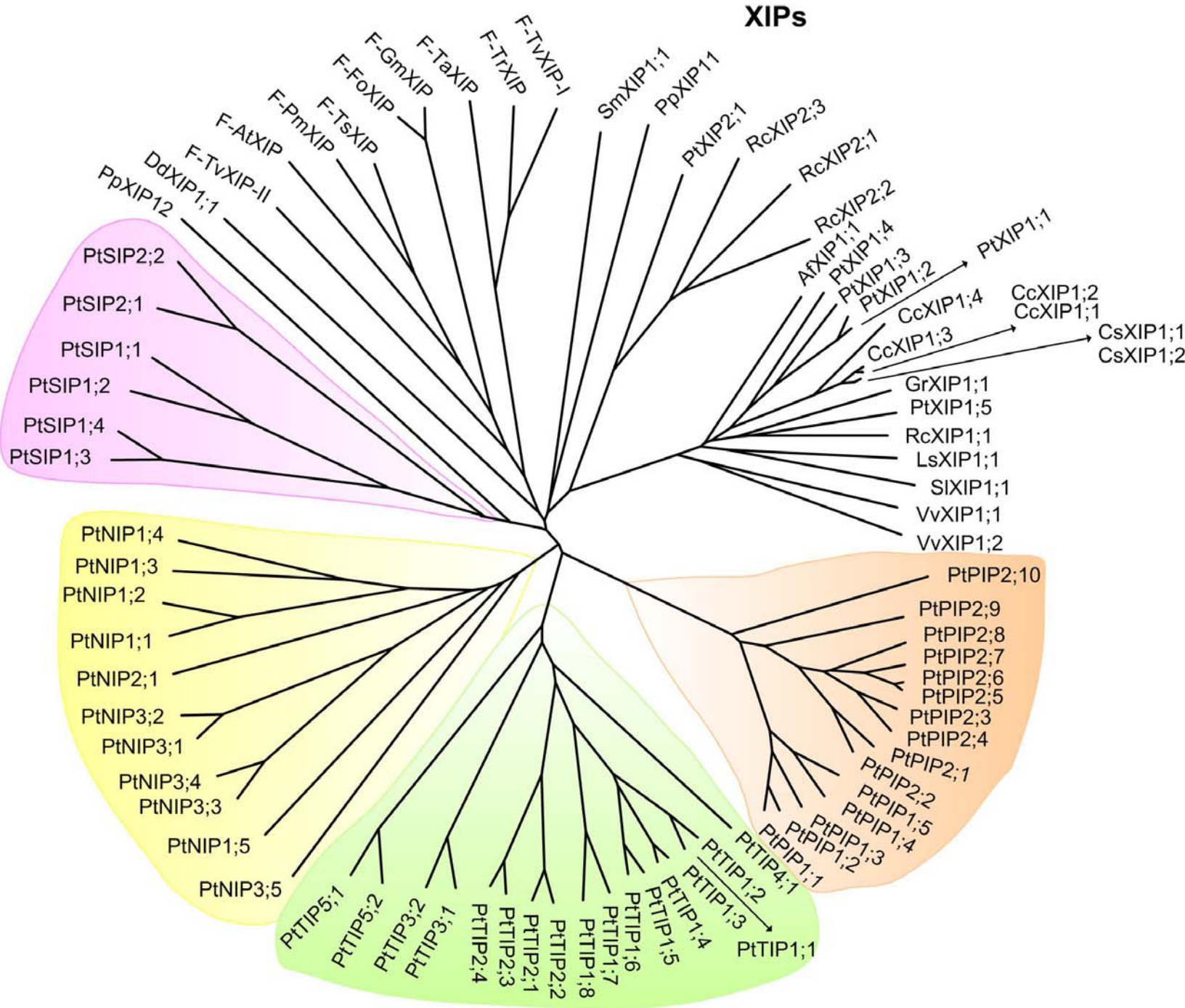

0.1

Supplement: Additional file 5 — Phylogenetic analysis of Populus MIPs and all XIPs. Phylogenetic analysis of all Populus MIPs is shown along with XIPs from the other dicot plants, fungi, moss and protozoa. Neighbor-Joining (NJ) method was used to create this rooted tree and NJ method used the multiple sequence alignment generated by T-COFFEE to generate the tree. Members of Populus XIP subfamily clustered with other XIPs and this group is separate from PtPIPs, PtTIPs, PtNIPs and PtSIPs. Background of non-XIP subfamilies are shown in different colors to distinguish from each other and from XIPs. [file 1471-2229-9-134-S5.PDF]
