# Supplementary material for: Genome-wide analysis of major intrinsic proteins in the tree plant Populus trichocarpa: Characterization of XIP subfamily of aquaporins from evolutionary perspective
Source: BMC Plant Biol. 2009 Nov 20;9:134. doi: 10.1186/1471-2229-9-134 (PMC2789079; doi:10.1186/1471-2229-9-134)
Supplement: Additional file 6 — Sequence alignment of loop C residues of all non-XIP plant MIPs. The sequence regions containing loop C are aligned for all non-XIP plant MIPs from Arabidopsis, Populus, rice and maize. Residues forming the last turn of H3 and the first turn of H4 are shown in gray background. All Gly and Pro residues are displayed in red and pink color respectively. The conserved residues within each subgroup are shown in green color. [file 1471-2229-9-134-S6.PDF]

Multiple sequence alignment of loop C residues of all PIPs

PIP1s

AtPIP1;1	GVVKG	FQPK-QYQALGGGANTVAHGYT	KGSGL
AtPIP1;2	GVVKG	FQPK-QYQALGGGANTIAHGYT	KGSGL
AtPIP1;3	GVVKG	FQPN-PYQTLGGGANTVAHGYT	KGSGL
AtPIP1;4	GVVKG	FQPT-PYQTLGGGANTVAHGYT	KGSGL
AtPIP1;5	GVVKG	FQPG-LYQTNGGGANVVAHGYT	KGSGL
OsPIP1;1	GVVKG	FQQG-LYMGNGGGANVVASGYT	KGDGL
OsPIP1;2	GVVKG	FQKG-LYETTGGGANVVAPGYT	KGDGL
OsPIP1;3	GVVKG	FQRG-LYMGSGGGANAVNPGYT	KGDGL
OsPIP1;4	GFVKG	FQQG-LFMGHGGGANVIATGYT	KGDGL
OsPIP1;5	GVVKG	FQKG-LYETTGGGANVVAPGYT	KGDRL
PtPIP1;1	GVVKG	FYGKKNYELLNGGANMVSPGYT	KGDGL
PtPIP1;2	GVVKG	FYGKTNYELHNGGANMVAHGYT	KGDGL
PtPIP1;3	GVVKG	LQGSHNYELQGGGANVVNHGYT	KGDGL
PtPIP1;4	GVVKG	FQKS-PYEILGGGANTVSTGYS	KGSGL
PtPIP1;5	AVVKA	FQKS-QYEMLGGGANTVSTGYA	KGSGL
ZmPIP1;1	GVVKG	FQQG-LYMGNGGRRNVVAPGYT	KGDGL
ZmPIP1;2	GVVKG	FQQG-LYMGNGGGANVVAPGYT	KGDGL
ZmPIP1;3	GVVKG	FQQG-LYMGNGGGANVVAPGYT	KGDGL
ZmPIP1;4	GVVKG	FQQG-LYMGNGGGANVVAPGYT	KGDGL
ZmPIP1;5	GVVKG	FQEG-LYMGAGGGANAVNPGYT	KGDGL
ZmPIP1;6	GVVKA	-FGSALYESAGGGANAVSPGYT	KGDGL

PIP2s

AtPIP2;1	GFVKA	FQSS-YYTRYGGGANSLADGYS	TGTGL
AtPIP2;2	GFVKA	FQSS-YYDRYGGGANSLADGYN	TGTGL
AtPIP2;3	GFVKA	FQSS-HYVNYGGGANFLADGYN	TGTGL
AtPIP2;4	GFVKA	FQSS-YYTRYGGGANELADGYN	KGTGL
AtPIP2;5	ALVKA	FQSA-YFTRYGGGANGLSDGYS	IGTGV
AtPIP2;6	GLVKV	FQST-YYNRYGGGANMLSDGYN	VGVGV
AtPIP2;7	GFVKA	FMKT-PYNTLGGGANTVADGYS	KGTAL
AtPIP2;8	GLVKA	FMMT-PYKRLGGGANTVADGYS	TGTAL
OsPIP2;1	GLVKA	FQSA-YFNRYGGGANTLAAGYS	KGTGL
OsPIP2;2	GLVKG	FQSS-YYARYGGGANELSDGYS	KGTGL
OsPIP2;3	GLVKG	FQSA-FYVRYGGGANELSDGYS	KGTGL
OsPIP2;4	ALVKG	FQSS-LYDRYGGGANELAAGYS	TGTGL
OsPIP2;5	ALVKG	FQSS-FYDRYGGGANELAAGYS	KGTGL
OsPIP2;6	GIVKG	IMKH-QYNANGGGANMVASGYS	TGTAL
OsPIP2;7	GIVKG	IMKR-PYDALGGGANTVSDGYS	AAGAL
OsPIP2;8	GLARA	MHGGGQYARHGGGANELAAGYS	AGAGV
PtPIP2;1	GLVKA	FMKP-YYNSLGGGANMVAPGYS	TGTAV
PtPIP2;10	LFVFL	FMYADEQQSSVGVVNVVSRNYS	KGAGL
PtPIP2;2	GLVKA	FMKE-NYNSLGGGANTVAMGYN	TGTAL
PtPIP2;3	GLVKA	FQKS-YYKKYGGGANTLADGFS	TGTGL
PtPIP2;4	GLVKA	FQKS-YYTKYNGGANVLADGYS	TGTGL
PtPIP2;5	GLVKA	FQKS-YYTKYGGGVNELATGFS	KGTGL
PtPIP2;6	GLVKA	FQKS-YYTKYGGGANELATGFS	KGTGL
PtPIP2;7	GLVKA	FQKS-YYTNYGGGANGLANGYS	KGTGL
PtPIP2;8	GLVKA	FQKS-YYNHYGGGANELQEGYN	KGTGL
PtPIP2;9	WMVMI	-LTGIHYDQAGGAVNVVAPGYS	KGTAL
ZmPIP2;1	GLVKA	FQSA-YFDRYGGGANSLASGYS	RGTGL
ZmPIP2;2	GLVKA	FQSA-YFDRYGGGANSLASGYS	RGAGL
ZmPIP2;3	GLVKG	FQSA-YYVRYGGGANELSDGYS	KGTGL
ZmPIP2;4	GLVKG	FQSA-YYVRYGGGANELSDGYS	KGTGL
ZmPIP2;5	GLVKG	FQSA-FYVRYGGGANELSAGYS	KGTGL
ZmPIP2;6	ALVKG	FQSG-FYARYGGGANEVSAGYS	TGTGL
ZmPIP2;7	GLAKG	FQKS-FYNRYGGGVNTVSDGYN	KGTAL

Multiple sequence alignment of loop C residues of all TIPs

TIP1s

AtTIP1;1	LILKF	ATGGLAVPAFGLSAGVG	VLNAF
AtTIP1;2	FLLSF	ATGGEPIPAFGLSAGVG	SLNAL
AtTIP1;3	LLLKV	STGGMETAAFSLSYGVT	PWNAV
OsTIP1;1	FLLRF	STGGLATGTFGLT-GVS	VWEAL
OsTIP1;2	LLLKI	ATGGAAVGAFSLSAGVG	AWNAV
PtTIP1;1	LLLKL	ATGGQETSAFALSSGVG	AWNAV
PtTIP1;2	LLLKL	ATGGLETSAFSLSSGVG	VWNAV
PtTIP1;3	LLLKF	ATGGLETPAFGLSSGVG	AWNAL
PtTIP1;4	LLLKF	STGGLETPAFGLSSGVG	AWNAV
PtTIP1;5	LLLKF	VTGGLETSAFALSTGVG	VWNAF
PtTIP1;6	LLLKF	TTGGLETSAFALSSGVG	VWNAF
PtTIP1;7	LLLKF	TPHYMTVSVFTLSPGVS	VWNAF
PtTIP1;8	LLLKY	TTHHMTVSVFTLSPGVT	VWNAF
ZmTIP1;1	FLLRF	STGGQATGTFGLT-GVS	VWEAL
ZmTIP1;2	LLLKI	ATGGAALGAFSLSAGVG	AMNAV

TIP2s

AtTIP2;1	FLLKY	VTGGLAVPTHSVAAGLG	SIEGV
AtTIP2;2	LLLVF	VTNGESVPTHGVAAGLG	AIEGV
AtTIP2;3	LLLVF	VTNGKSVPTHGVSAGLG	AVEGV
OsTIP2;1	LLLKF	VTHGKAIPTHGVA-GIS	ELEGV
OsTIP2;2	VLVQF	CT-GVATPTHGLS-GVG	AFEGV
OsTIP2;3	LLCSS	PPTDRLAIPTHAIA-GIS	EIEGM
PtTIP2;1	YLLKV	ATGGLAVPIHSVAAGVG	AIEGV
PtTIP2;2	YLLKV	VTGGLAVPIHSVAAGVG	AIEGV
PtTIP2;3	LLLKV	VTSAEGIPTHGVASGMS	AIEGV
PtTIP2;4	LLLKF	ATSAESIPTHGVASGMS	AVEGV
ZmTIP2;1	LLLGF	VTHGKAIPTHAVA-GIS	ELEGV
ZmTIP2;2	LLLRF	VTHGKAIPTHGVSGGTT	ELEGV
ZmTIP2;3	FLLQY	VTHGQAIPTHGVS-GIS	EIEGV

TIP3s

AtTIP3;1	LLLRL	TTNGMRPVGFRLASGVG	AVNGL
AtTIP3;2	LLLRL	ATNGLRPVGFHVASGVS	ELHGL
OsTIP3;1	LLLRL	TTGGMRPPGFALASGVG	DWHAV
OsTIP3;2	LLLRL	ASGGMRPMGFTLGHRIH	ERHAL
PtTIP3;1	LLLRL	VTNGMIPAGFHVQSEVG	EVHGL
PtTIP3;2	LLLRL	VTNGMRPVGFHVQSGVG	EVHGL
ZmTIP3;1	LLLRL	ATGGMRPPGFALASGVG	DWHAV

TIP4s

AtTIP4;1	FLLSY	LTGGMGT-PVHTLASGVS	YTQGI
OsTIP4;1	ILLRY	LTGGMAT-PVHTLGSGIG	PMQGL
OsTIP4;2	LLLRC	LTGGAAT-PVHALADGVG	PVQGV
OsTIP4;3	LLLAA	LTGGEEAVPVHAPAPGVG	AARAV
PtTIP4;1	FLLKY	LTGGLAT-SVHTLASGMD	YLQGV
ZmTIP4;1	VLLRF	LSGGMVT-PVHALGRGIS	PMQGL
ZmTIP4;2	ILLRY	LSGGMVT-PVHALGAGIR	PMQGL
ZmTIP4;3	FLLRW	LTGGLAT-PVHALAEGVG	PLQGV
ZmTIP4;4	LLLAF	LAVADSGVPVHALGAGVG	ALRGV

TIP5s

AtTIP5;1	LVLKV	TVMEQHVPIYKIAGEMT------------	GFGAS
OsTIP5;1	LVLHY	ISAGQAVPTTRIAVEMT------------	GFGAG
PtTIP5;1	IFLKV	ATVGQHVPTNTIAEEMT------------	GFGAS
PtTIP5;2	IFLKV	TTVGQQFPTYTIAEEMT------------	GFGAS
ZmTIP5;1	LSLNL	FSAGEEVPTTRIAVAMTGFGGAVLEGVLT	FLLVY

Multiple sequence alignment of loop C residues of all NIPs

NIP1s

AtNIP1;1	ATLRL	LFGLDHDVCSG------KHDVFIGSSPVGS	DLQAF
AtNIP1;2	ATLRL	LFGLDQDVCSG------KHDVFVGTLPSGS	NLQSF
OsNIP1;1	GTLRL	-------MFGG------RHEHFPGTLPAGS	DVQSL
OsNIP1;2	LTLRV	-------VFGGGGGGARGEHLFFGTTPAGS	MAQAA
OsNIP1;3	AALRA	-------LFGG------APEHFFGTAPAGS	DVQSL
OsNIP1;4	VVLRL	-------MFGG------RHAPVPATLPGGA	HAQSL
OsNIP1;5	LTLRV	------VVFGGGGS-ARGEHLFLGTTPAG-	MAQAA
PtNIP1;1	GTIRL	-------LFQG------DQDHFTGTMPAGS	NLQSF
PtNIP1;2	GTIRL	-------IFQG------KQDHFTGTMPAGS	DLQSF
PtNIP1;3	GTLSL	-------LFS------VTDEAYFGTIPVGP	DIRSF
PtNIP1;4	GTLAL	-------ALD------VTPEAFFGTVPVGS	DGQSL
PtNIP3;5	YVGSS	-------VYG------VKTELMTTR-PAIG	CSSAF
ZmNIP1;1	GTLRL	-------MFGG------RHEHFPGTLPTGS	EVQSL

NIP2s

AtNIP2;1	ATLRL	LFDLNNDVCSKKHDVFLGSS-PSGS	DLQAF
OsNIP2;1	FVLKA	-------VIH--PVDVIGTTTPVGP	HWHSL
OsNIP2;2	FVLRA	-------VLY--PIEVLGTTTPTGP	HWHAL
PtNIP2;1	FTLKV	-------LLH--PIRNVGTTSPSGT	AVQAL
ZmNIP2;1	FVLKA	-------VLH--PIAVLGTTTPTGP	HWHSL
ZmNIP2;2	FVLKA	-------VLH--PIAVIGTTTPSGP	HWHAL

NIP3s

AtNIP3;1	AVLRL	VFHLDDDVCSLKGDVYVGTY--PSNS--	NTTSF
OsNIP3;1	FALKG	-------VFH---PFLSGGVTVPDPTIS	TAQAF
OsNIP3;2	FAVKG	-------MYH---PVNPGIVTVP--KVG	TVEAF
OsNIP3;3	FAVKG	-------LYH---PVNPGIVTVP--NVG	TVEAF
OsNIP3;4	AAVDG	-------IFH---PASRGWM--------	VKELI
OsNIP3;5	FLAKG	------ VYRPARPAVMATV-PAA-GVG	AGEAF
PtNIP3;1	FALKG	-------IFH---PVMGGGVTVP--SGG	YGQAF
PtNIP3;2	FALKV	-------IFH---PMMGGGVTVP--SGG	HGQAF
PtNIP3;3	FALKG	-------VFH---PFMSGGVTVP--SVS	TGQAF
PtNIP3;4	FALKG	-------VFH---PFMSGGVTVP--SVS	TGQAF
PtNIP3;5	YVGSS	-------VYG---VKTELMTTRP--AIG	CSSAF
ZmNIP3;1	FALKG	-------VFH---PFLSGGVTVPDATVS	TAQAF

Other NIPs

AtNIP4;1	LTLRL	MFKVTPEA--FFGTT-PADS---	PARAL
AtNIP4;2	LTLRL	MFNVTPKA--FFGTT-PTDS---	SGQAL
OsNIP4;1	LSVNA	--VMRPRHDHFYGTA-PVVVHG-	TRLPF
AtNIP5;1	FALKG	--VFHP--FMSGGVTIP--SVS	LGQAF
AtNIP6;1	FALKA	--VFEP--TMSGGVTVP--TVG	LSQAF
AtNIP7;1	LVGVS	---VYGVNADIMATK-PALS--	CVSAF

Multiple sequence alignment of loop C residues of all SIPs

SIP1s

AtSIP1;1	LAIME	FIPEKYKHMI-GGPSLQVD	VHTGA
AtSIP1;2	ITIME	MIPEKYKTRIGGKPSLQFG	AHNGA
OsSIP1;1	MAISE	LMPEQYKHML-GGPSLKVD	LHTGA
PtSIP1;1	LAILE	VMPLQYKHML-GGPTLQVD	LQTGG
PtSIP1;2	LAIME	VMPVQYKHML-GGPTLQVD	LHTGG
PtSIP1;3	MAIRG	VMPKHYRQLLKGGPSLRVD	LHTGA
PtSIP1;4	MAITE	VMPKQYRYVLRGGPSLKVD	LHTGA
ZmSIP1;1	LAISE	LMPAQYKHTL-AGPSLKVD	PHTGA
ZmSIP1;2	LAISE	LMPAQYRHML-GGPSLKVD	PHTGA

SIP2s

AtSIP2;1	KHIIH	VFPEIGKGPKLNVA	IHHGA
OsSIP2;1	KLIRA	ALPKVGKGAPLSVG	VHHGA
PtSIP2;1	RLFID	TFPEIGLGPRLTVD	IHKGA
PtSIP2;2	RLLID	TFPEIGRGPRLNVD	IHKGA
ZmSIP2;1	KLIQV	TFPNVGKGARLSVG	AHHGA
