# Supplementary material for: Genome-wide analysis of major intrinsic proteins in the tree plant Populus trichocarpa: Characterization of XIP subfamily of aquaporins from evolutionary perspective
Source: BMC Plant Biol. 2009 Nov 20;9:134. doi: 10.1186/1471-2229-9-134 (PMC2789079; doi:10.1186/1471-2229-9-134)
Supplement: Additional file 7 — Sequence alignment of loop D residues of all non-XIP plant MIPs. Multiple sequence alignment of loop D residues of all non-XIP plant MIPs. Residues forming the last turn of H4 and first turn of H5 are displayed in gray background. Acidic (Asp and Glu) and basic (Arg, Lys and His) residues are shown in blue and red respectively. [file 1471-2229-9-134-S7.PDF]

# Multiple sequence alignment of loop D residues of all PIPs

## PIP1s

| | | | |
|---|---|---|---|
| AtPIP1;1 | VFSAT | DAKRNARDSHVPI-------- | LAPLP |
| AtPIP1;2 | VFSAT | DAKRNARDSHVPI-------- | LAPLP |
| AtPIP1;3 | VFSAT | DAKRSARDSHVPI-------- | LAPLP |
| AtPIP1;4 | VFSAT | DAKRSARDSHVPVWTPLLVPI | LAPLP |
| AtPIP1;5 | VFSAT | DAKRSARDSHVPI-------- | LAPLP |
| OsPIP1;1 | VFSAT | DAKRNARDSHVPI-------- | LAPLP |
| OsPIP1;2 | VFSAT | DAKRNARDSHVPI-------- | LAPLP |
| OsPIP1;3 | VFSAT | DAKRNARDSHVPI-------- | LAPLP |
| OsPIP1;4 | VFSAT | DAKRNARDSHVPI-------- | LAPLP |
| OsPIP1;5 | VFSAT | DAKKNARDSHVPI-------- | LAPLP |
| PtPIP1;1 | VFSAT | DAKRSARDSHVPI-------- | LAPLP |
| PtPIP1;2 | VFSAT | DAKRSARDSHVPI-------- | LAPLP |
| PtPIP1;3 | VFSAT | DAKRNARDSHVPI-------- | LAPLP |
| PtPIP1;4 | VFSAT | DAKRSARDSHVPV-------- | LAPLP |
| PtPIP1;5 | VFSAT | DAKRNARDSHVPI-------- | LAPLP |
| PtPIP2;1 | VFSAT | DPKRSARDSHIPV-------- | LAPLP |
| ZmPIP1;1 | VFSAT | DAKRRARDSHVPI-------- | LAPLP |
| ZmPIP1;2 | VFSAT | DAKRNARDSHVPI-------- | LAPLP |
| ZmPIP1;3 | VFSAT | DAKRNARDSHVPI-------- | LAPLP |
| ZmPIP1;4 | VFSAT | DAKRNARDSHVPI-------- | LAPLP |
| ZmPIP1;5 | VFSAT | DAKRSARDSHVPI-------- | LAPLP |
| ZmPIP1;6 | VFSAT | DAKRTARDSHVPA-------- | LAPLP |

## PIP2s

| | | | |
|---|---|---|---|
| AtPIP2;1 | VFSAT | DPKRSARDSHVPV | LAPLP |
| AtPIP2;2 | VFSAT | DPKRNARDSHVPV | LAPLP |
| AtPIP2;3 | VFSAT | DPKRNARDSHVPV | LAPLP |
| AtPIP2;4 | VFSAT | DPKRNARDSHVPV | LAPLP |
| AtPIP2;5 | VFSAT | DPKRSARDSHVPV | LAPLP |
| AtPIP2;6 | VFSAT | DPKRNARDSHIPV | LAPLP |
| AtPIP2;7 | VFSAT | DPKRSARDSHIPV | LAPLP |
| AtPIP2;8 | VFSAT | DPKRSARDSHVPV | LAPLP |
| OsPIP2;1 | VFSAT | DPKRNARDSHVPV | LAPLP |
| OsPIP2;2 | VFSAT | DPKRNARDSHIPV | LAPLP |
| OsPIP2;3 | VFSAT | DPKRNARDSHVPV | LAPLP |
| OsPIP2;4 | VFSAT | DPKRNARDSHVPV | LAPLP |
| OsPIP2;5 | VFSAT | DPKRNARDSHVPV | LAPLP |
| OsPIP2;6 | VFSAT | DPKRNARDSHVPV | LAPLP |
| OsPIP2;7 | VFSAT | DPKRTARDSFIPV | LVPLP |
| OsPIP2;8 | VFSAT | DPKRKARDSHVPV | LAPLP |
| PtPIP2;10 | VFSAT | DPKRNARDSHVPV | LAPLP |
| PtPIP2;2 | VFSAT | DPKRSARDSHVPV | LAPLP |
| PtPIP2;3 | VFSAT | DPKRSARDSHVPV | LAPLP |
| PtPIP2;4 | VFSAT | DPKRSARDSHVPV | LAPLP |
| PtPIP2;5 | VFSAT | DPKRNARDSHVPV | LAPLP |
| PtPIP2;6 | VFSAT | DPKRNARDSHVPV | LAPLP |
| PtPIP2;7 | VFSAT | DPKRNARDSHVPV | LAPLP |
| PtPIP2;8 | VFSAT | DPKRNARDSHVPV | LAPLP |
| PtPIP2;9 | VLAAT | DPKRMARDSHVPV | LAPLP |
| ZmPIP2;1 | VFSAT | DPKRNARDSHVPV | LAPLP |
| ZmPIP2;2 | VFSAT | DPKRNARDSHVPV | LAPLP |
| ZmPIP2;3 | VFSAT | DPKRSARDSHVPV | LAPLP |
| ZmPIP2;4 | VFSAT | DPKRSARDSHVPV | LAPLP |
| ZmPIP2;5 | VFSAT | DPKRNARDSHVPV | LAPLP |
| ZmPIP2;6 | VFSAT | DPKRNARDSHVPV | LAPLP |
| ZmPIP2;7 | VFSAT | DPKRNARDSHVPV | LAPLP |

## Multiple sequence alignment of loop D residues of all TIPs

### TIP1s

| | | | |
|---|---|---|---|
| AtTIP1;1 | VYATA | IDPKNGSLGT | IAPIA |
| AtTIP1;2 | VYATA | VDPKNGSLGT | IAPIA |
| AtTIP1;3 | VYATA | VDPKKGDIGI | IAPLA |
| OsTIP1;1 | VYATA | VDPKKGSLGT | IAPIA |
| OsTIP1;2 | VYATA | VDPKKGDLGV | IAPIA |
| PtTIP1;1 | VYATA | VDPKKGDIGI | IAPIA |
| PtTIP1;2 | VYATA | VDPKRGDIGI | IAPIA |
| PtTIP1;3 | VYATA | VDPKKGNLGI | IAPIA |
| PtTIP1;4 | VYATA | VDPKKGNLGI | IAPIA |
| PtTIP1;5 | VYATA | IDPKKGNLGI | IAPIA |
| PtTIP1;6 | VYATA | VDPKKGNLGI | IAPIA |
| PtTIP1;7 | VYATA | IDAKKGDVGV | IAPLA |
| PtTIP1;8 | VYATA | IDPKKGDVGV | IAPLA |
| ZmTIP1;1 | VYATA | VDPKKGSLGT | IAPIA |
| ZmTIP1;2 | VYATA | VDPKKGDLGV | IAPIA |

### TIP2s

| | | | |
|---|---|---|---|
| AtTIP2;1 | VYATA | ADPKKGSLGT | IAPLA |
| AtTIP2;2 | VYATA | ADPKKGSLGT | IAPIA |
| AtTIP2;3 | VYATA | ADPKKGSLGT | IAPIA |
| OsTIP2;1 | VYATA | ADPKKGSLGT | IAPIA |
| OsTIP2;2 | VYATA | ADPKKGSLGT | IAPIA |
| OsTIP2;3 | VYATA | ADPKKGSLGT | VAPMA |
| PtTIP2;1 | VYATA | ADPKKGSLGT | IAPIA |
| PtTIP2;2 | VYATA | ADPKKGSLGT | IAPIA |
| PtTIP2;3 | VYATA | ADPKKGSLGI | IAPIA |
| PtTIP2;4 | VYATA | ADPKKGSIGI | IAPIA |
| ZmTIP2;1 | VYATA | ADPKKGSLGT | IAPIA |
| ZmTIP2;2 | VYATA | ADPKKGSLGT | IAPIA |
| ZmTIP2;3 | VYATA | ADPKKGSLGT | IAPMA |

### TIP3s

| | | | |
|---|---|---|---|
| AtTIP3;1 | VYSTL | IDPKRGSLGI | IAPLA |
| AtTIP3;2 | VYSTA | IDPKRGSIGI | IAPLA |
| OsTIP3;1 | YYATV | IDPKRGHVGT | IAPLA |
| OsTIP3;2 | VYATA | VDRRSGGG-D | IAPLA |
| PtTIP3;1 | VYATA | IDPKRGSLGI | IAPLA |
| PtTIP3;2 | VYATA | LDPKRGSLGI | IAPLA |
| ZmTIP3;1 | YYATV | IDPKRGHVGT | IAPLA |

### TIP4s

| | | | |
|---|---|---|---|
| AtTIP4;1 | VYATI | VDPKKGSLDG | FGPLL |
| OsTIP4;1 | VYATI | LDPRSSVP-G | FGPLL |
| OsTIP4;2 | ICATI | LDPRRAAPPG | TGPLL |
| OsTIP4;3 | VYATV | VDRRRAVG-A | LGPLL |
| PtTIP4;1 | VYATI | VDPKKGSIDG | LGPML |
| ZmTIP4;1 | TYAMI | LDPRSQVR-A | IGPLL |
| ZmTIP4;2 | TYAMI | LDPRSQVR-T | IGPLL |
| ZmTIP4;3 | IYATI | LDPRKLLP-G | AGPLL |
| ZmTIP4;4 | VYATV | VDPRRAVG-G | MGPLL |

### TIP5s

| | | | |
|---|---|---|---|
| AtTIP5;1 | VFTAS | -DPR--RG------LPLAV | VGPIF |
| OsTIP5;1 | VHVAG | -DPRGGGFGGRKGPAATA- | LGALV |
| PtTIP5;1 | VYAAG | -DPR--RG------SLGA- | IGPLA |
| PtTIP5;2 | VYAAG | -DPR--RS------SLGA- | IGPLA |
| ZmTIP5;1 | GGDGK | ---R--EF------AATA- | LGALA |

# Multiple sequence alignment of loop D residues of all NIPs

## NIP1s

| | | | |
|---|---|---|---|
| AtNIP1;1 | ISGVA | TDNRAKLNIGTKCCNIQIGE | LAGLA |
| AtNIP1;2 | ISGVA | ------------TDNRAIGE | LAGLA |
| OsNIP1;1 | ISGVA | ------------TDNRAIGE | LAGLA |
| OsNIP1;2 | VSGVA | ------------TDNRAIGE | LAGLA |
| OsNIP1;3 | VSGVA | ------------TDNRAIGE | LAGLA |
| OsNIP1;4 | IMAVA | ------------TDDQAVGH | MAGVA |
| OsNIP1;5 | VSSVA | ------------TDNRAIGE | LAGLA |
| PtNIP1;1 | ISGVA | ------------TDNRAIGE | LAGLA |
| PtNIP1;2 | ISGVA | ------------TDNRAIGE | LAGLA |
| PtNIP1;3 | ISGVA | ------------TDNRAIGE | LAGIA |
| PtNIP1;4 | ISGVS | ------------TDDRAVGD | LAGIA |
| PtNIP1;5 | ICGVA | ------------TDPRASKD | LSGVA |
| ZmNIP1;1 | ISGVA | ------------TDNRAIGE | LAGLA |

## NIP2s

| | | | |
|---|---|---|---|
| AtNIP2;1 | VCAVT | TTKRTTEE | LEGLI |
| OsNIP2;1 | TLAVA | TDTRAVGE | LAGLA |
| OsNIP2;2 | TCAVA | TDSRAVGE | LAGLA |
| PtNIP2;1 | TSAVA | TDTKAVGE | LAGIA |
| ZmNIP2;1 | TLAVA | TDTRAVGE | LAGLA |
| ZmNIP2;2 | TCAVA | TDSRAVGE | LAGLA |

## NIP3s

| | | | |
|---|---|---|---|
| AtNIP3;1 | ISAVA | TDKRATGS | FAGIA |
| OsNIP3;1 | VTAVA | TDTRAVGE | LAGIA |
| OsNIP3;2 | ITALA | TDPNAVKE | LIAVA |
| OsNIP3;3 | ITALA | TDPNAVKE | LIAVA |
| PtNIP3;1 | VTAVA | TDTRAVGE | LAGIA |
| PtNIP3;2 | VTAVA | TDTRAVGE | LAGIA |
| PtNIP3;3 | VTAVA | TDTRAVGE | LAGIA |
| PtNIP3;4 | VTAVA | TDTRAVGE | LAGIA |
| PtNIP3;5 | AASLT | SQSRSIGP | LSGFL |
| ZmNIP3;1 | VTAVA | TDTRAVGE | LAGIA |

## NIP4s

| | | | |
|---|---|---|---|
| AtNIP4;1 | ISGVA | TDNRAVGE | LAGIA |
| AtNIP4;2 | ISGVA | TDSRATGE | LAGIA |
| OsNIP4;1 | IATVA | TDGTAGKT | VGGIA |

## Other NIPs

| | | | |
|---|---|---|---|
| AtNIP5;1 | VTAVA | TDTRAVGE | LAGIA |
| AtNIP6;1 | VTAVA | TDTRAVGE | LAGIA |
| AtNIP7;1 | ASALH | CDFVQLGN | LTGFV |

## Multiple sequence alignment of loop D residues of all SIPs

| | | | |
|---|---|---|---|
| **SIP1s** | | | |
| AtSIP1;1 | VLLII | LRGPRRLL | AKTFL |
| AtSIP1;2 | VLLII | LRGPRKLL | AKTFL |
| OsSIP1;1 | VLWII | VKGPRNPI | VKTWM |
| PtSIP1;1 | VLVII | LKGPRSSL | VQAWF |
| PtSIP1;2 | VLVII | LKGPRNPL | VQTLF |
| PtSIP1;3 | LHFLL | LKGPKNVV | LKVWF |
| PtSIP1;4 | LHFVL | LKGPKNFV | LKVWL |
| ZmSIP1;1 | VLWVI | VKGPRNVI | LKTLL |
| ZmSIP1;2 | VLLII | VKGPRNPI | IKTWM |
| **SIP2s** | | | |
| AtSIP2;1 | SMGLT | RKIPGSFF | MKTWI |
| OsSIP2;1 | SVTLK | KKEMKGFF | MKTWI |
| PtSIP2;1 | SLGLA | RKIPGSFF | MKTWI |
| PtSIP2;2 | SLGLA | RKIPGSFF | MKTWI |
| ZmSIP2;1 | SVTLK | KKEMKSFF | KTWIT |
