# Supplementary material for: Genome-wide analysis of major intrinsic proteins in the tree plant Populus trichocarpa: Characterization of XIP subfamily of aquaporins from evolutionary perspective
Source: BMC Plant Biol. 2009 Nov 20;9:134. doi: 10.1186/1471-2229-9-134 (PMC2789079; doi:10.1186/1471-2229-9-134)
Supplement: Additional file 8 — Group conservation of small and weakly polar conserved interfacial residues in Populus MIPs. Conservation of small and weakly polar residues (Gly, Ala, Ser, Thr, Cys) as a group is reported for all Populus MIPs and also individually for the subfamily members. Conservation was found out for 17 positions using structure-based sequence alignment of MIP sequences. The residues at these 17 positions occur at the helix-helix interface of the transmembrane helix bundle. [file 1471-2229-9-134-S8.PDF]

**Table S3: Group conservation of small and weakly polar residues at the helix-helix interface of PtMIPs**

| Residue[a] | All families[b,c] | PtPIPs[c] | PtTIPs[c] | PtNIPs[c] | PtSIPs[c] | PtXIPs[c] |
|---|---|---|---|---|---|---|
| T48 | S, T(91) ***(95)*** | T(100) ***(100)*** | T(82) ***(82)*** | A(100) ***(100)*** | T(67),S(33) ***(100)*** | T(100) ***(100)*** |
| T55 | G(45), T(25), S, A ***(80)*** | T(87),G,S ***(100)*** | G(88) ***(88)*** | G(64),A,T ***(91)*** | G(33),S(33) ***(67)*** | --- |
| A78 | A(69), S, C, T ***(91)*** | A(100) ***(100)*** | A(88),S ***(100)*** | A(55),S(27),C ***(100)*** | T, A ***(33)*** | S(67), A ***(83)*** |
| G82 | G(51), A(41) ***(91)*** | G(100) ***(100)*** | A(88), G ***(100)*** | G(100) ***(100)*** | A(33) ***(33)*** | A(83) ***(83)*** |
| A103 | A(76), S, T ***(87)*** | A(100) ***(100)*** | A(100) ***(100)*** | A(64),S(36) ***(100)*** | T(33),A(33) ***(67)*** | A ***(17)*** |
| G107 | G(58),A(25),S,T ***(98)*** | G(100) ***(100)*** | G(100) ***(100)*** | A(91),T ***(100)*** | S(67),T(33) ***(100)*** | A(67),S ***(83)*** |
| G129 | G(87),A ***(100)*** | G(100) ***(100)*** | G(82),A ***(100)*** | G(64),A(36) ***(100)*** | G(100) ***(100)*** | G(100) ***(100)*** |
| A130 | S(56),A(33),G,T ***(100)*** | A(80),T,S ***(100)*** | S(100) ***(100)*** | S(82),A ***(100)*** | S(50),G(33),A ***(100)*** | A(50),G(33),S ***(100)*** |
| G133 | A(55),G(45) ***(100)*** | G(100) ***(100)*** | A(100) ***(100)*** | A(100) ***(100)*** | G(83),A ***(100)*** | G(83),A ***(100)*** |
| T172 | T(82),G,S,A ***(100)*** | T(100) ***(100)*** | T(94),A ***(100)*** | T(64),S(36) ***(100)*** | T(83),A ***(100)*** | G(67),T(33) ***(100)*** |
| S181 | A(42),S(27),G,T ***(84)*** | S(93),A ***(100)*** | A(100) ***(100)*** | A(45),G(45),S ***(100)*** | G(33) ***(33)*** | T ***(17)*** |
| G203 | G(87),A,S ***(100)*** | G(100) ***(100)*** | G(100) ***(100)*** | G(100) ***(100)*** | A(67),S(33) ***(100)*** | G(83),A ***(100)*** |
| S226 | S(60),A,C,T ***(93)*** | S(100) ***(100)*** | S(65),A ***(88)*** | S(64),T(36) ***(100)*** | A(67) ***(67)*** | C(100) ***(100)*** |
| G248 | G(73),A,C,S ***(98)*** | G(100) ***(100)*** | G(100) ***(100)*** | A(45),S(27),G ***(91)*** | C(67),A(33) ***(100)*** | G(100) ***(100)*** |
| G252 | G(87),A ***(98)*** | G(100) ***(100)*** | G(100) ***(100)*** | G(91) ***(91)*** | G(100) ***(100)*** | A(100) ***(100)*** |
| A253 | A(60),G,C,S,T ***(98)*** | A(100) ***(100)*** | G(65),A,S ***(100)*** | A(82),T ***(91)*** | A(83),T ***(100)*** | C(83),S ***(100)*** |
| A256 | A(71),G ***(87)*** | A(100) ***(100)*** | A(100) ***(100)*** | G(82),A ***(91)*** | A(100) ***(100)*** | --- |

[a]Residue numbers correspond to that of spinach aquaporin SoPIP2;1 (PDB ID: 1Z98)

[b]Group-based conservation is reported for all the five *populus* MIP subfamilies based on structure-based sequence alignment of 55 *Populus* MIPs

[c]Small and weakly polar interfacial residues in Populus MIP sequences and their conservation (if it exceeds 25%) are given. If the conservation is less than 25%, only the residues are given. For each position, group conservation of all the five residues (Gly, Ser, Thr, Ala and Cys) is shown in bold and italics.
